# Hypoxia-primed monocytes/macrophages enhance postinfarction myocardial repair

**DOI:** 10.7150/thno.63642

**Published:** 2022-01-01

**Authors:** Yu Zhu, Wenjuan Yang, Hailong Wang, Fuqin Tang, Yun Zhu, Qiong Zhu, Ruiyan Ma, Zhao Jian, Yingbin Xiao

**Affiliations:** 1 Department of Cardiovascular Surgery, the Second Affiliated Hospital, Army Medical University, 400037, Chongqing, China.; 2 Vascular Injury and Repair Laboratory, the Second Affiliated Hospital, Army Medical University, 400037, Chongqing, China.; 3 Department of Ultrasound, the Second Affiliated Hospital, Army Medical University, 400037, Chongqing, China.

**Keywords:** hypoxia, myocardial infarction, monocytes/macrophages, phenotypic transition, AMPKα2

## Abstract

**Background:** Oxygen supplementation in myocardial infarction (MI) remains controversial. Inflammation is widely believed to play a central role in myocardial repair. A better understanding of these processes may lead to the design of novel strategies complementary to MI treatment.

**Methods:** To investigate the role of hypoxia in inflammation and myocardial repair after acute MI, we placed MI mice in a tolerable mild hypoxia (10% O_2_) chamber for 7 days and then transferred the mice to ambient air for another 3 weeks.

**Results:** We found that the cumulative survival rate of the MI mice under hypoxia was significantly higher than that under oxygen supplementation. Hypoxia promoted postinfarction myocardial repair. Importantly, we found that hypoxia modulated the phenotypic transition of blood monocytes from pro-inflammatory to pro-reparative in a timely manner, leading to the subsequent discontinuation of inflammation in myocardial tissues and promotion of myocardial repair post-MI. Specifically, cultured bone marrow-derived macrophages (BMDMs) primed by hypoxia *in vitro* exhibited improved reparative capacities and differed from M_1_ and M_2_ macrophages through the AMPKα2 signaling pathway. The deletion of AMPKα2 in monocytes/macrophages prevented the phenotypic transition induced by hypoxia and could not promote myocardial repair after MI when transplanted into the myocardium.

**Conclusions:** Taken together, our work demonstrates that hypoxia promotes postinfarction myocardial repair by modulating the blood monocyte/macrophage phenotypic transition from pro-inflammatory to pro-reparative in a timely manner through the AMPKα2 signaling pathway. Hypoxia priming might be an attractive translational strategy for MI treatment by amplifying immune cells during early inflammation and subsequent resolution and repair.

## Introduction

A large population of inflammatory cells, including neutrophils and monocytes, are mobilized to the myocardium during acute myocardial infarction (AMI) 1. This process occurs in a time-dependent manner, and the rapid infiltration of neutrophils is followed by an influx of monocytes 2. Monocytes/macrophages play different roles during early inflammatory and late repair processes 3, 4. However, the specific environmental cues that induce monocyte/macrophage polarization and promote cardiac repair after AMI remain unclear 5.

Oxygen supplementation has been a cornerstone of supportive care in patients suspected of having AMI for more than a century and is widely endorsed by international guidelines 6, 7. However, clinical studies have increasingly confirmed that oxygen supplementation has no beneficial effects when administered to ST-elevation myocardial infarction (STEMI) patients with no hypoxemia 8, 9. Moreover, some studies proposed that the administration of oxygen supplementation therapy to acutely ill patients could be toxic and increase mortality and morbidity 10.

Recently, studies have confirmed that hypoxia is a major regulatory factor responsible for maintaining the proliferative capacity of cardiomyocytes in neonatal mice and inducing the regeneration of cardiomyocytes in adult mice 11-13. Hypoxia can promote macrophage phenotypic transition to a specific type or the M_2_ phenotype in solid tumors 14. Therefore, the role of hypoxia in heart recovery after MI seems to be protective, and further studies are needed to confirm this phenomenon.

Adenosine 5'-monophosphate (AMP)-activated protein kinase (AMPK) is a highly conserved, key regulator of intracellular energy homeostasis. The catalytic α subunit has the following two isoforms: α1 is localized to the cytosol, while α2 can translocate to the nucleus 15. AMPKα2 has been shown to regulate the stability of hypoxia-inducible factor-1α (HIF-1α) and neutrophil survival, thereby determining further myeloid cell recruitment and repair potential after hindlimb ischemia 16. AMPK is upregulated under hypoxic conditions and promotes cardiomyocyte adaptation to chronic hypoxia 17. However, the role of AMPK in modulating the functions of monocytes/macrophages under AMI is still largely unknown.

In the present study, we investigated the role of hypoxia in cardiac repair after MI and the mechanism involved.

## Methods

### Study Approval

The Institutional Animal Care and Use Committee of Army Medical University (Shapingba District, Chongqing, China) approved all animal care and experimental procedures prior to the commencement of this study (AMUWEC2020666). The authors declare that all supporting data are available within the article.

### Experimental Protocols, Animals, and Surgical Procedures

Eight- to twelve-week-old male C57BL/6 mice from Charles River Laboratories (Stock No: 219) were used for the *in vivo* and *in vitro* experimental protocols. The mice were subjected to general anesthesia with isoflurane, and then, a thoracotomy was performed. A 7-0 silk suture was used to permanently ligate the LAD. The animals were allowed to recover under aseptic conditions and received analgesic medication (buprenorphine, 0.1 mg/kg s.c.) to reduce postoperative pain.

CX3CR1-GFP BM-derived macrophages were isolated from CX3CR-1^GFP^ knock-in mice from The Jackson Laboratory (Stock No: 005582). CX3CR1-GFP BM-derived macrophages (1×10^6^) or phosphate buffered saline (PBS) were injected at 3 different sites along the infarct border zone at a final volume of 10 µL in each site. Clodronate liposomes (dichloroethylene diphosphonate [Cl2MDP]) were injected through the caudal vein to deplete systemic macrophages. All operated mice were anesthetized with an intraperitoneal injection of sodium pentobarbital (80 mg/kg) and then euthanized by cervical dislocation.

### Cardiac Functional Measurements

The mice were anesthetized by isoflurane. Transthoracic echocardiography was performed to evaluate the cardiac function. The measurements of the LV end-systolic dimension and LV end-diastolic dimension were obtained through two-dimensional short and long axes. The ejection fraction and fraction shortening were calculated (Table S1, S2). After measuring the heart function by echocardiography, the mice were immediately euthanized by cervical dislocation, and the hearts were collected and randomly divided for different assays in further experiments.

### Tissue Harvesting

Heparin (10 IU/g) was administered through an intraperitoneal (i.p.) injection 30 min before anesthesia. Then, the mice were deeply anesthetized with isoflurane. The abdominal cavity was opened, and the inferior vena cava was separated. Then, 500 μL of saturated potassium chloride solution (KCl) were injected through the inferior vena cava to induce heart arrest in the diastolic phase. Next, the heart was excised and washed with PBS on ice, and the connective tissue was quickly removed. The heart was fixed in 4% paraformaldehyde for paraffin-cut sectioning or embedded in OCT compound and stored at -80 °C until sectioning. Sectioning commenced from the apical part, and each heart slice was cut at a 6 μm thickness. Serial slices were obtained from 12 equally distant (300 μm) regions originating from the heart apex.

### Histology, Immunohistochemistry, and Immunocytochemistry

Masson's trichrome staining. Paraffin-cut sections were stained according to the manufacturer's protocol and imaged under a microscope. Morphometric analyses of the infarcted tissue were performed using ImageJ software. Infarct size measurements were performed with the assistance of MI Quant software.

Picrosirius red staining and polarized light microscopy. Paraffin-cut heart sections were stained according to the manufacturer's protocol (Abcam). The sections were digitized using an Olympus slide scanner. Fibrosis was quantified as a picrosirius red-positive area. The red-orange collagen area was compared with the green area in fibrosis.

Immunohistochemistry. Frozen slices were fixed with 4% PFA and blocked with bovine serum albumin (BSA). The primary antibodies are listed in Table S3. To detect apoptotic cardiomyocytes, a TdT dUDP nick-end labeling assay (TUNEL, Roche) was performed according to the manufacturer's protocol, and the samples were stained with cTnT and DAPI.

### Bone Marrow-derived Monocyte (BMDM) Isolation and Macrophage Differentiation

Femurs were isolated from 8- to 12-week-old C57BL/6 mice. The bone marrow (BM) was isolated, flushed with PBS, and filtered through a 70-μm mesh. A red blood cell (RBC) lysate was used to remove RBC. The bone marrow cells were washed, centrifuged and resuspended for plating. After 3 days, the media were exchanged. The BMDMs were incubated overnight between days 7 and 8 to polarize toward normoxic macrophages. On day 3 after the media change, for the hypoxia treatment, the macrophages were induced in a 1% O_2_ and 5% CO_2_ cell incubator. The positive control for the hypoxic macrophages was stimulated with CoCl_2_. On day 7, the medium was changed. New growth medium containing 10% FBS and 100 ng/mL LPS with 50 ng/mL IFN-γ was used for the M_1_ activation, while a medium containing 10% FBS with 10 ng/mL IL-4 and 10 ng/mL IL-13 was used for the M_2_ activation. Polarized activated macrophages were harvested for the gene expression examination after 24 h of stimulation. A flow cytometry analysis of these cells was performed 48 h after the stimulation.

### Macrophage Migration Assay

Migration of macrophages through 8-μm pores were assessed using a Transwell® Cell Culture chamber (Corning Costar, NY, USA). New medium supplemented with 15% fetal bovine serum (FBS) was added to the lower chamber and 1 × 10^4^ cells in 100 μL of serum-free medium were added to the upper chamber and incubated for 24 h at 37 °C. After removing the non-migrated cells from the upper surface of the membrane, the cells that moved through the pores were fixed in 4% paraformaldehyde and stained with 1% Giemsa blue (Sigma-Aldrich). The membranes were photographed and cells were counted.

### Cell Isolation and Flow Cytometry

The hearts were perfused with 4 °C PBS buffer. The infarct zone and border zone were separated and gently minced into 1-mm^3^ granules and digested using Collagenase II. The digested material was filtered through a 200-mesh screen filter and centrifuged at 1000 rpm for 5 min at 4 °C. The red blood cells were lysed and then resuspended in FACS buffer. The suspended cells were incubated with conjugated antibodies (Table S4) for 30 min at 4 °C protected from light. Peripheral blood was collected through the abdominal aorta. Leukocytes were washed and resuspended in FACS buffer. All conjugated antibodies used in this study are listed in Table S4. The analysis was performed using a BD FACS Verse flow cytometer and FlowJo 10.0 software.

### Protein and RNA Isolation

Protein. At the appropriate time point, the macrophages were harvested and rinsed with PBS. Cell lysis buffer was used to lyse the macrophages on ice for 30 min. It was necessary to scrape the culture plates on ice. The lysates were centrifuged, and the supernatant containing the sample protein was collected. The concentrations were measured using a BCA assay.

RNA. The infarct zone and border zone were separated from the heart and gridded in liquid nitrogen. TRIzol was added to the mouse heart tissue, which was stored for a long time until use at -80 °C. The macrophages were washed twice and collected for the RNA isolation using TRIzol (1×10^6^ cells). The following steps were performed according to the manufacturer's protocol.

### Quantitative RT-PCR

SYBR Green was used to compare the gene profile levels under different processing conditions following normoxia and hypoxia. cDNA was first synthesized from mRNA using a PrimeScript^TM^ RT reagent kit according to the manufacturer's protocol. The resulting cDNA was standardized across the samples depending on the sample concentration. Then, the gene profile was amplified over the course of 30 cycles and analyzed by the ∆∆CT method. Primer sets predesigned for the following genes were purchased from Invitrogen. The sequences are listed in Table S5 and S6.

### Macrophage Gene Array

Library construction and sequencing. The total RNA was extracted from the cells/tissues using TRIzol according to the manufacturer's protocol, and ribosomal RNA was removed using a Ribo-Zero™ kit. Fragmented RNA was subjected to first- and second-strand cDNA synthesis, followed by adaptor ligation and enrichment with a low cycle according to the instructions of the NEBNext® Ultra™ RNA Library Prep Kit for Illumina. The purified library products were evaluated using an Agilent 2200 TapeStation and Qubit®2.0 (Life Technologies, USA). The libraries were paired-end sequenced (PE150, sequencing reads were 150 bp) at Guangzhou RiboBio Co., Ltd. (Guangzhou, China) using an Illumina HiSeq 3000 platform.

### Western Blot Analysis

Equal amounts of protein samples were loaded for gel electrophoresis according to the manufacturer's protocol. Briefly, the proteins were separated through SDS-PAGE and then transferred to polyvinylidene fluoride membranes. The membranes were incubated overnight with primary antibodies, followed immediately by secondary antibody incubation. A Western blot imaging system was used to show chemiluminescence to reveal immunoreactivity. The gray value was analyzed by ImageJ software.

### Statistical Analysis

The data are expressed as the mean ± standard deviation (SD) or number (percentage) and were analyzed by GraphPad Prism 8.0 software and SPSS 26.0 software. Unpaired t-test, Mann-Whitney test, analysis of variance (ANOVA) followed by Sidak's multiple comparisons or Kruskal-Wallis test followed by Dunn's posttest for correction were used to evaluate the differences between the groups. The sample capacity estimation was performed using SPSS 26.0 software. The Kaplan-Meier method was used to estimate the survival curves, and the log-rank test was performed to examine the comparisons between the groups. The differences were considered statistically significant only when the *P*-value was less than 0.05.

## Results

### Hypoxia Promotes Murine Cardiac Repair and Survival after MI

We first evaluated whether mice could tolerate 10% O_2_ conditions for a long period. We found that the survival rate of the MI mice under long-term hypoxia conditions was significantly lower than that of the mice under ambient air conditions (Figure S1A). The heart weight did not significantly differ between the two groups of mice (Figure S1B), but the lung weight was increased under hypoxia (Figure S1C). Meanwhile, the tibia length and food intake were reduced in the MI mice under the hypoxic condition (Figure S1D-E). Even though we provided equivalent food to the MI mice that received ambient air and the MI mice that underwent hypoxia, the reductions in body weight were more obvious in the hypoxic MI mice 28 days after MI (Figure S1F).

**Figure 1:**
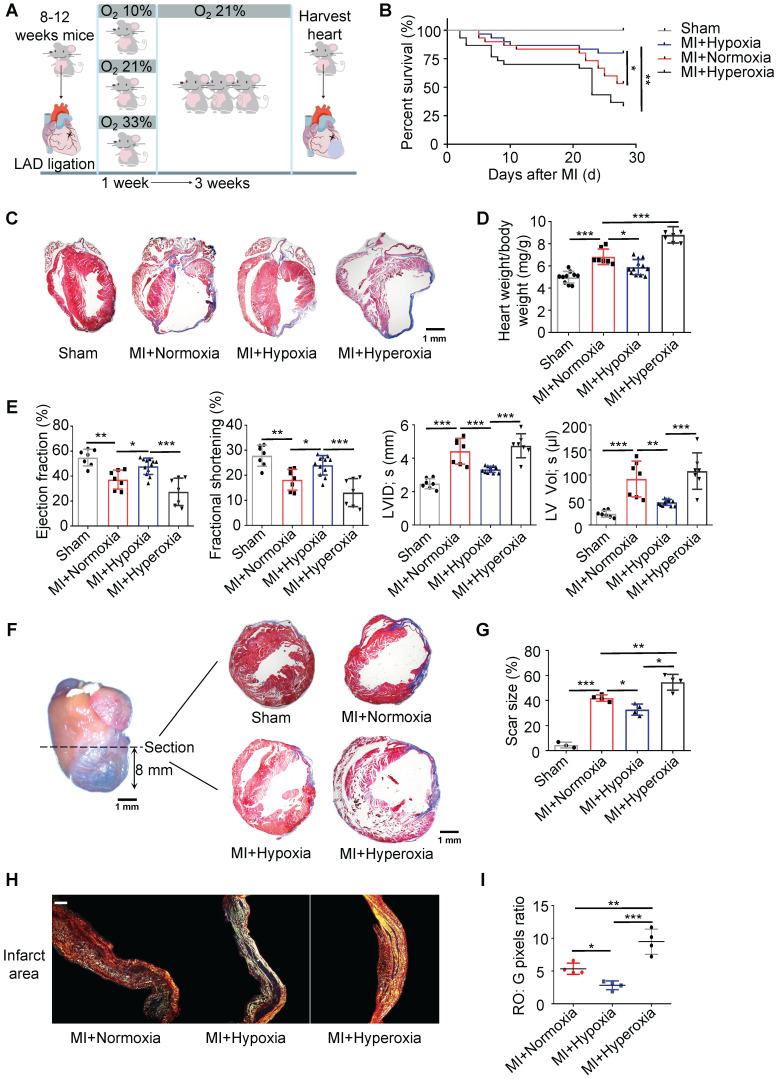
** Hypoxia promotes postinfarction myocardial repair in mice. (A)** Mice were exposed to 10% O_2_ after LAD ligation-induced MI, which was then maintained for 7 days, and then, the mice were subjected to normoxia (21% O_2_) for 21 days. Hearts were harvested 28 days after MI. **(B)** Cumulative survival curve (Kaplan-Meier survival plot) of MI mice under hypoxia, normoxia and hyperoxia and sham-operated mice (n = 30 for the MI + hypoxia group, n = 30 for the MI + normoxia mice group, n = 30 for the MI + hyperoxia group, and n = 10 for the sham-operated group).** (C)** Representative results of trichrome staining of longitudinal heart sections after MI. Scale bar: 1 mm. **(D)** Heart weight-to-body weight (HW/BW) ratio in sham, MI + normoxic, MI + hypoxic and MI + hyperoxic mice. **(E)** Left ventricular ejection fraction (LVEF), fractional shortening (LVFS), left ventricular internal diameter at end-systole (LVID, s) and left ventricular volume at end-systole (LV Vol; s) were measured by echocardiography and are presented. **(F)** Schematic representation of left ventricle (LV) sampling 8 mm from the apical region to the section. Trichrome staining of the transverse section is presented. (n = at least 3 per group). Scale bar: 1 mm. **(G)** Quantification of the fibrotic area relative to the myocardium in transverse sections. **(H)** Representative images of infarct areas stained with picrosirius red and visualized under polarized light. Scale bar: 100 μm.** (I)** Quantification of picrosirius red polarized light analysis in the infarct areas of MI mice. Dot-plots represent data from individual mice. Statistical significance was determined by log-rank test (**B**), ANOVA followed by Sidak's multiple comparisons test (**D**,** E**) or Kruskal-Wallis test followed by Dunn's posttest to correct for multiple comparisons (**G**,** I**). *P < 0.05, **P < 0.01 and ***P < 0.001 compared to the corresponding control.

Thus, we chose to expose mice to 10% O_2_ for 7 days immediately after MI (Figure 1A). The body weights and food intake were comparable between the MI mice exposed to hypoxia and those exposed to ambient air (Figure S2A-B). To study the toxic effect of hyperoxemia on postinfarction myocardial repair, we subjected another group of mice to 33% O_2_ to mimic hyperoxia for 7 days and then transferred these mice to ambient air for another 21 days (Figure 1A). On day 28, the survival rate of the hypoxia-treated mice (approximately 80%) was significantly higher than that of the ambient air-treated mice (approximately 53%, p < 0.05), while the survival rate of the hyperoxia-treated mice (approximately 33%) was significantly lower (Figure 1B, p < 0.01) than that of the normoxia-treated MI mice. Four weeks after MI, 11 of 30 ambient air-treated mice (36.67%), 4 of 30 hypoxia-treated mice (13.33%), and 13 of 30 hyperoxia-treated mice (43.33%) had died with signs of heart failure (Figure S2C). Rupture deaths were observed less frequently in our results and were not significantly affected by different conditions (Figure S2D). Moreover, the hypoxia-treated mice demonstrated significantly less cardiac compensatory hypertrophy, which was measured as the heart weight-to-body weight ratio, than the ambient air-treated mice (Figure 1C-D). The heart dimensions and function were evaluated, and compared to the normoxic MI mice, the left ventricular (LV) dimension was smaller, and heart function was better in the hypoxic MI mice (Figure 1E and Table S1). Sequential heart sections from the apex to the ligation point showed that the thickness of the LV free wall was less reduced in the hypoxia-treated mice compared with that in the ambient air-treated mice. The scar size and LV compensatory hypertrophy were changed in the same manner as the thickness of the LV free wall (Figure 1F-G and Figure S3A-B). Collagen deposition was measured by picrosirius red under polarized light microscopy, and the tightly packed versus loosely packed fiber ratio (red-orange: green) in the infarcts and border zones in the hypoxic MI mice was significantly lower than that in the normoxic MI mice (Figure 1H-I and Figure S4A-C).

### Hypoxia Mitigates Cardiomyocyte Apoptosis and Promotes Myocardial Repair after MI

**Figure 2:**
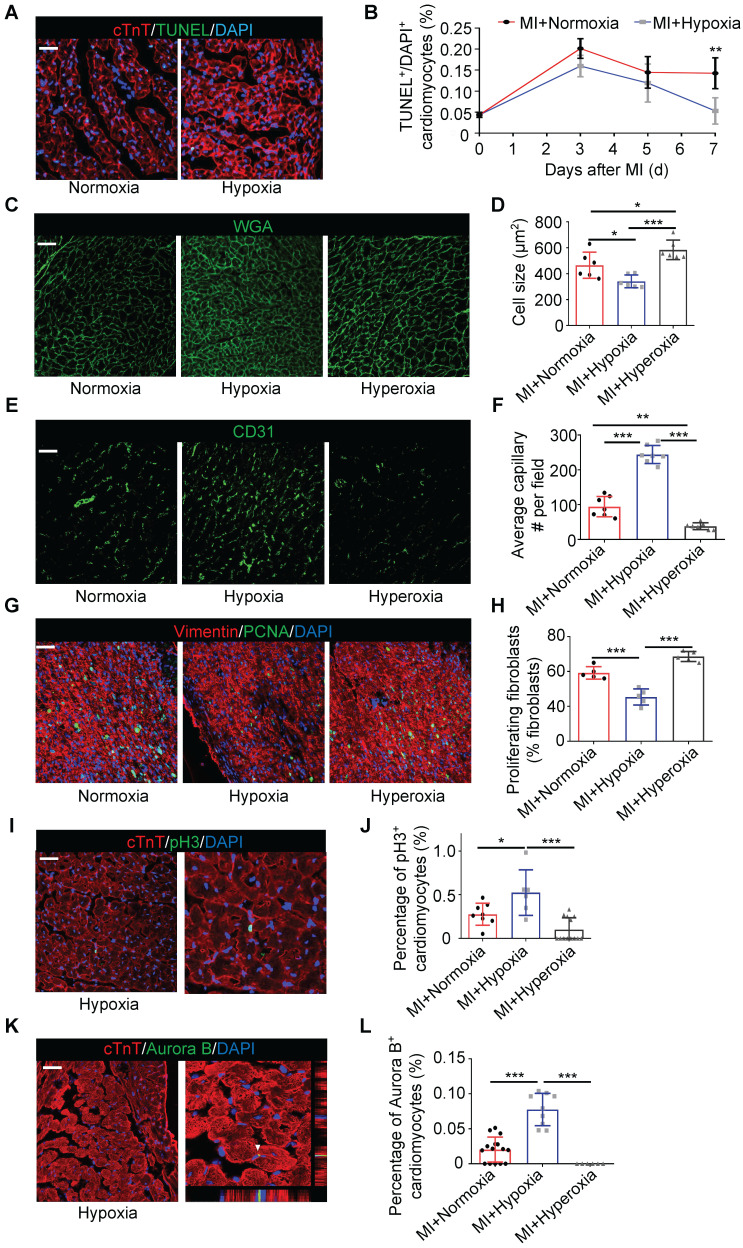
** Hypoxia mitigated cardiomyocyte apoptosis, induced angiogenesis and promoted cardiomyocyte proliferation. (A)** Representative immunofluorescence images of TUNEL-stained heart tissue from the border zones of normoxia- and hypoxia-treated MI mouse hearts. **(B)** Quantitative assessment of cardiomyocytes in A and similar images revealed reduced TUNEL positivity in the hypoxia-treated MI mouse hearts at different time points (n = 3 at each time point). **(C)** Representative immunofluorescence images of wheat germ agglutinin (WGA)-stained heart tissue in the border zone. **(D)** Pooled analyses of the cardiomyocyte size.** (E)** Representative immunofluorescence images of CD31-stained heart tissue in the border zone. **(F)** Quantification of CD31 positivity surrounding cardiomyocytes. **(G)** Representative images of coimmunofluorescent staining of proliferating cardiac fibroblasts in the infarct area of mice under normoxia, hypoxia and hyperoxia 7 days postinfarction (MI). **(H)** Quantification of proliferating cardiac fibroblasts in the infarct area of mice. **(I)** Representative immunofluorescence images of coimmunostaining with anti-pH3 and anti-cTnT antibodies in the border zones of MI hearts. **(J)** Quantification of pH3+ cardiomyocytes after MI in the hypoxia and normoxia groups.** (K)** Representative immunofluorescence images of coimmunostaining with anti-Aurora B and anti-cTnT antibodies in the border zones of MI hearts. **(L)** Quantification of Aurora B+ cardiomyocytes after MI in the hypoxia and normoxia groups. Dot-plots represent data from individual mice. Statistical significance was determined by ANOVA followed by Sidak's multiple comparisons test. *P < 0.05, **P < 0.01 and ***P < 0.001 compared to the corresponding MI+normoxia. Scale bar: 50 μm.

We further examined cell death in the border zone at different time points after MI. There was no significant difference in the number of TUNEL-positive cardiomyocytes at the border zone during the first 5 days after MI between the hypoxic and normoxic mice. The number of TUNEL-positive cardiomyocytes was strikingly reduced in the MI mice treated with hypoxia on the 7th day after MI (Figure 2A-B). The cardiomyocyte size, which was determined by wheat germ agglutinin (WGA) staining, in the hypoxic group was significantly smaller than that in the normoxic or hyperoxic group after MI (Figure 2C-D). The vessel density in the border zone in the MI mice subjected to hypoxia was also higher than that in the MI mice subjected to normoxia or hyperoxia (Figure 2E-F and Figure S5A-B). There was a significant trend toward decreased fibroblast proliferation in the infarct area in the hypoxic group compared to the normoxic or hyperoxic group after MI (Figure 2G-H and Figure S4D). As hypoxia promotes cardiomyocyte re-entry into the proliferation cycle in adult mice, we measured the expression of phosphorylated histone H3 Ser10 (pH3), which is a mitosis marker in the heart, on day 28 after MI. The number of pH3-positive cardiomyocytes was increased in the MI mice under hypoxia conditions (Figure 2I-J and Figure S5C). Aurora B kinase-positive cardiomyocytes, which are markers of cytokinesis, were also increased in the hypoxia-treated mice (Figure 2K-L and Figure S5D).

### Hypoxia Modulates the Ratio of Pro-inflammatory to Pro-reparative Monocytes/Macrophages in Blood and Injured Myocardial Tissue

**Figure 3:**
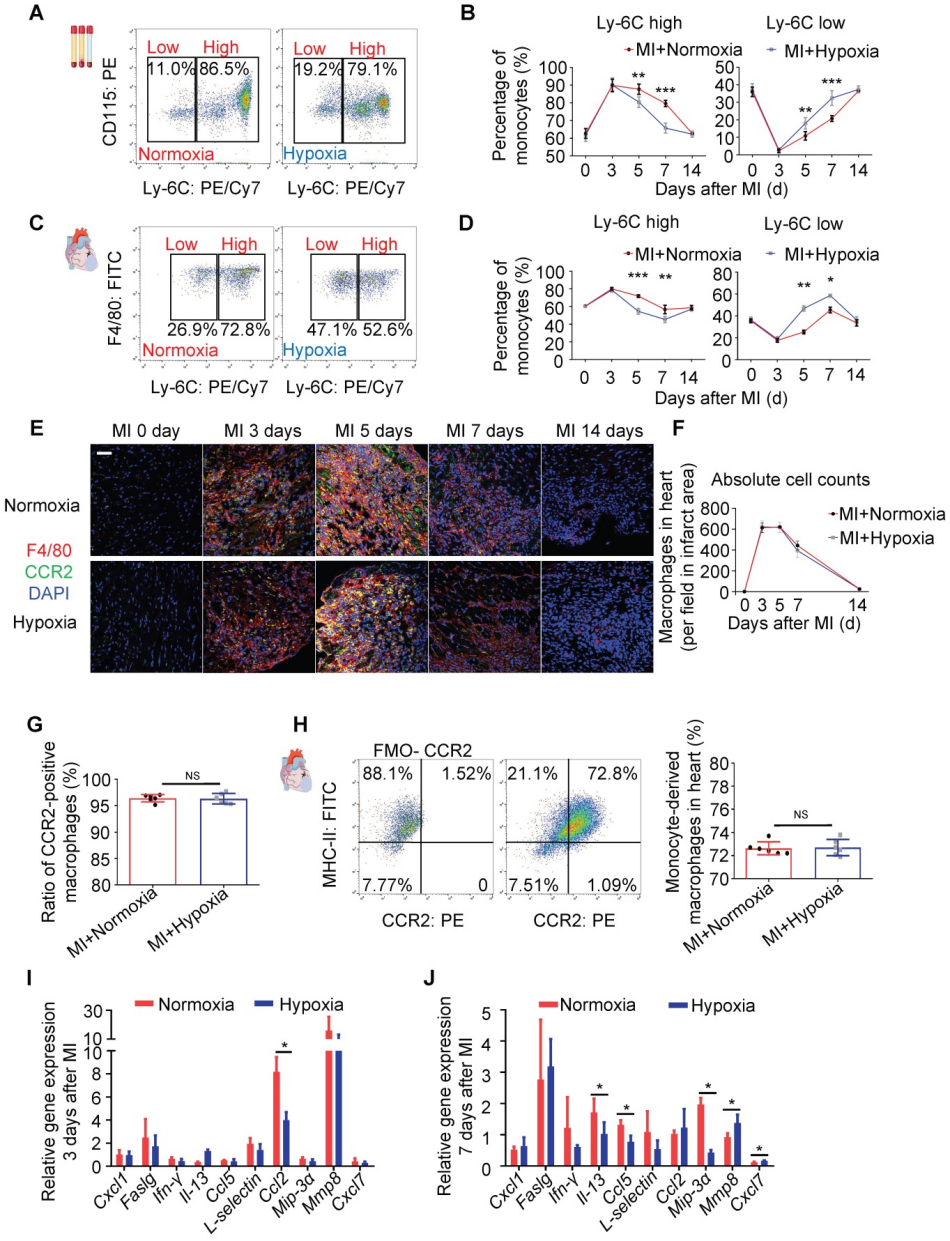
** Systemic hypoxia promoted monocyte/macrophage phenotypic transitions. (A)** Representative flow cytometric analysis of blood monocytes 5 days after MI under normoxia or hypoxia. **(B)** Ratios of Ly6C^high^ and Ly6C^low^ blood monocytes among all blood monocytes (n=3 per group at each time point). **(C)** Representative flow cytometric analysis of tissue monocytes/macrophages 5 days after MI under normoxia or hypoxia. **(D)** Ratios of Ly6C^high^ and Ly6C^low^ tissue monocytes among all monocytes (n=3 per group at each time point). **(E)** Representative immunofluorescence images of coimmunostaining with anti-CCR2 and anti-F4/80 antibodies within the infarct zone over 14 days in mice after LAD ligation. Scale bar: 50 μm. **(F)** Cell counts of macrophages in the infarct area in each field at various time points after MI in the normoxia or hypoxia group (n = 3 per group). **(G)** No difference was observed in the ratio of CCR2-positive macrophages in the MI mouse hearts between the hypoxia and normoxia groups. **(H)** Pooled flow cytometry data from infarcted mouse tissue revealed no difference in the CCR2+ macrophage population between the normoxia- and hypoxia-treated MI hearts. **(I)** Protein expression of inflammatory cytokines in the infarct zones of hearts treated with hypoxia 3 days after LAD ligation (n = 3 per group). **(J)** Protein expression of inflammatory cytokines in the infarct zones of hearts treated with hypoxia 7 days after LAD ligation (n = 3 per group). Dot-plots represent data from individual mice. Statistical significance was determined by a Mann-Whitney test (**B, D, F, I, J**) or unpaired *t*-test (**H**). ^NS^*P* > 0.05, *P < 0.05, **P < 0.01 and ***P < 0.001 compared to the corresponding MI+normoxia group.

To determine whether the systemic inflammatory reaction of monocytes was upregulated under hypoxia in our study, we analyzed the inflammatory subtype of monocytes by staining harvested blood cells for lymphocyte antigen 6 complex locus C (Ly6C). The gating strategy for monocyte identification in blood is shown in Figure S6A. Ly6C^high^ monocytes are generally considered a pro-inflammatory subtype, while Ly6C^low^ monocytes are considered a subtype associated with the resolution of inflammation and the propagation of repair 14. There was no difference in the ratio of Ly6C^high^ cells during the first to third days after MI between the mice exposed to ambient air and those exposed to hypoxia. However, the ratio of Ly6C^low^ cells in the hypoxic group was strikingly higher than that in the ambient air group on the 5th day after MI (Figure 3A-B), and representative flow cytometric results at different time points are shown in Figure S6B. Similarly, the ratio of tissue Ly6C^low^ monocytes/macrophages was also significantly increased on the 5th day after MI in heart tissue from the mice under hypoxia (Figure 3C-D). The gating strategy and representative flow cytometric results at different time points are shown in Figure S7A-B. These results are consistent with the apoptosis level measured in injured myocardium as there was no significant difference in the first 5 days, but the apoptosis level was reduced after 7 days of hypoxia (Figure 2B).

Furthermore, we found that there was no significant change in the numbers of monocytes in the blood and monocytes/macrophages in heart tissue subjected to hypoxia after MI (Figure S6C and Figure S7C). Meanwhile, the ratio of Ly6C^low^ splenic monocytes in total blood monocytes in the hypoxic group was higher than that in the ambient air group on the 5th day after MI (Figure S8A-C), which is consistent with the changes we observed in blood (Figure 3A-B). Moreover, we detected the effect of hypoxia on myelopoiesis in the spleen and bone marrow and found that systemic hypoxia did not exert a major impact on myelopoiesis in the bone marrow and spleen (Figure S8D-I).

F4/80^+^ macrophage accumulation peaked at approximately 5 days after MI and then rapidly decreased on approximately the 7th day after MI, and the hypoxia treatment had no impact on this process (Figure 3E-F). To further characterize the macrophage population, we detected M_1_ and M_2_ macrophage infiltration into the myocardium over time after MI. We found that hypoxia had no impact on the number of M_1_ and M_2_ macrophages in the infarct area (Figure S9A-D). C-C motif chemokine receptor 2 (CCR2) was used to distinguish the monocyte-derived macrophages (CCR2^+^) from the resident macrophages (CCR2^-^) 18. Laser confocal microscopy and flow cytometry showed that CCR2^+^ macrophages were the overwhelming cell type in the early stage after MI (Figure 3E-H and Figure S10A).

Next, we measured the expression of cytokines associated with inflammation in the ischemic zone in the MI hearts. On the third day after MI, hypoxia significantly inhibited chemokine (C-C motif) ligand 2 (CCL2) expression, which was associated with inflammation (Figure 3I) 19. On the 7^th^ day after MI, interleukin-13 (IL-13), chemokine (C-C motif) ligand 5 (CCL5) and chemokine (C-C motif) ligand 20 (CCL20, also known as MIP-3a) were decreased in the hypoxia-treated MI hearts, while matrix metalloproteinase-8 (MMP8) and chemokine (C-X-C motif) ligand 7 (CXCL7) were upregulated by hypoxia (Figure 3J) 20. Accordingly, as higher Ly6C^low^ content would be consistent with efficacious inflammation resolution, we therefore examined dynamic changes of inflammatory cytokines, like IL-1β, Il-1α, IL-6, IL-4, Nos2, Arg-1 and IL-10. We found the level of IL-1β, Il-1α, IL-6 and Nos2 declined in hypoxia-treated group after MI (Figure S11A). In the contrast, Arg-1 and IL-10, increased in hypoxia-treated group after MI on day 5 and day 7 (Figure S11B). These results confirm that hypoxia modulates the ratio of reparative monocytes/macrophages in both the blood and injured myocardial tissue during the late resolution of inflammation and propagates repair with no significant effects on the pro-inflammatory functions of monocytes/macrophages during the early period.

### Systemic Depletion of Macrophages Using Clodronate Reduces the Efficacy of Hypoxia after MI

We tested whether the systemic depletion of macrophages could recapitulate the benefits of hypoxia-induced cardioprotection to establish the importance of macrophages in mediating hypoxia-induced cardiac protection. We administered clodronate (dichloromethylene diphosphonate [Cl2MDP]) liposomes immediately after MI via the caudal vein. Clodronate effectively reduced the spleen and heart macrophage populations after MI (Figure S12A-B). Clodronate attenuated the benefits of hypoxia after MI as follows: we observed no differences in the morphology of the left ventricle between the normoxia- and hypoxia-treated groups (Figure S12C-D). Echocardiography revealed LV aneurysms and mural thrombi in both groups (Figure S12E). No obvious differences were observed in the left ventricular ejection fraction (LVEF) and left ventricular internal diameter at end-systole (LVID, s) measured using echocardiography (Figure S12F). An even greater scar size was detected in the hypoxia-treated group compared with that in the normoxia-treated group (Figure S12G-H).

### Hypoxia-Primed Monocytes/Macrophages Promote Postinfarction Myocardial Repair* In Vivo*

**Figure 4:**
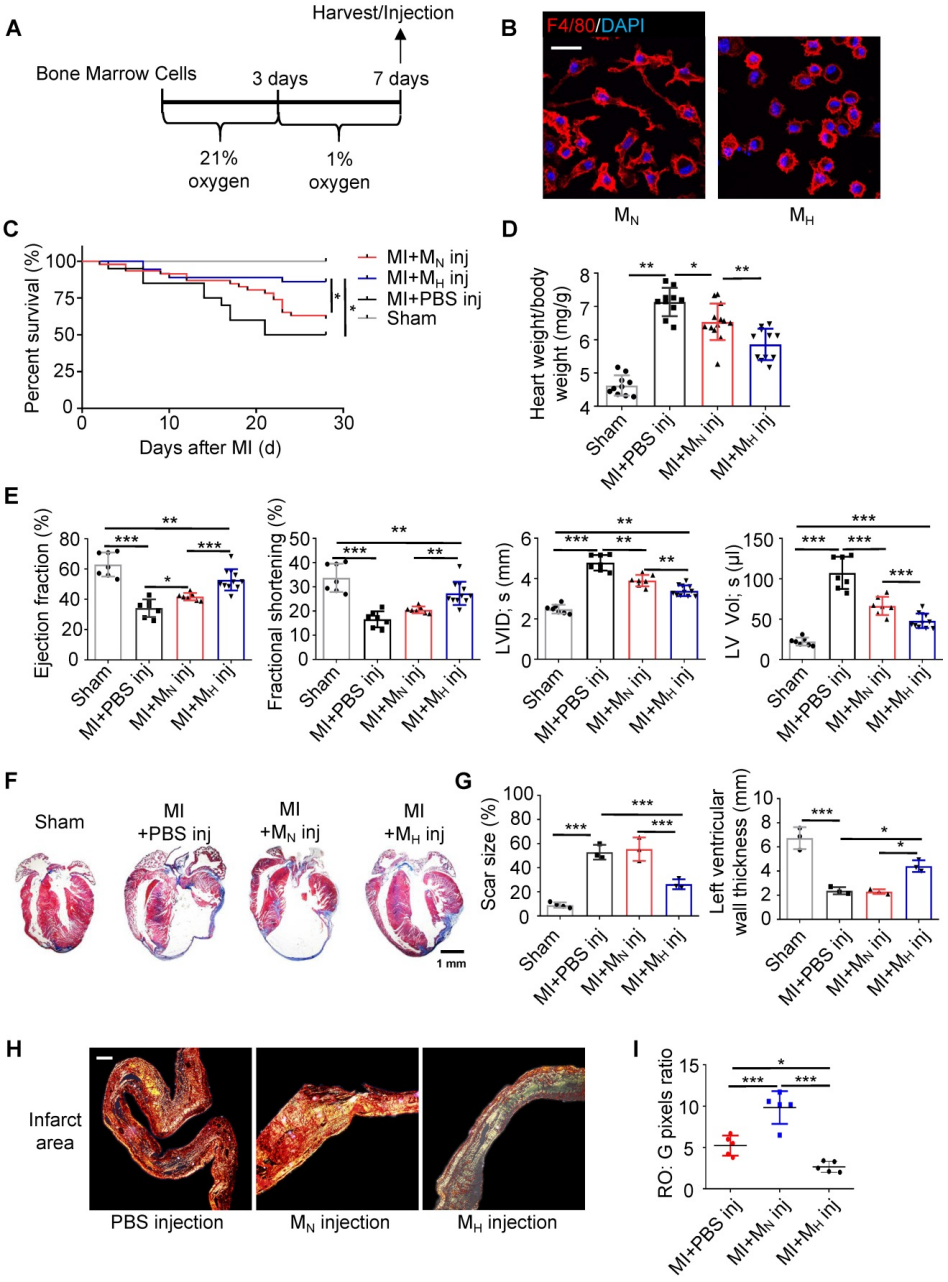
**Hypoxia-primed monocytes/macrophages promote postinfarction myocardial repair* in vivo*. (A)** Schematic protocol of intramyocardial injection. The animals were monitored for 28 days. **(B)** Representative laser confocal microscopy images of M_N_- and M_H_-polarized macrophages. Scale bar: 50 μm. **(C)** Cumulative survival curve (Kaplan-Meier survival plot) of MI mice after macrophage transplantation and sham-operated mice (n = 46 for MI followed by M_N_ macrophage transfer, n = 36 for MI followed by M_H_ macrophage transfer, n = 20 for MI followed by vehicle transfer, and n = 10 for sham-operated mice). **(D)** The heart weight-to-body weight (HW/BW) ratio was significantly decreased in the MI mice following the M_H_ macrophage transfer. **(E)** Left ventricular ejection fraction (LVEF), fractional shortening (LVFS), left ventricular internal diameter at end-systole (LVID, s) and left ventricular volume at end-systole (LV, Vol, s) were measured by echocardiography and are presented. **(F)** Trichrome staining of longitudinal sections showed enlarged hearts after MI. Scale bar: 1 mm. **(G)** Quantification of the fibrotic area and LV thickness relative to the myocardium in transverse sections demonstrated a significant decrease in scar formation in the hypoxia-treated MI mice and an increase in scar formation in the hyperoxia-treated mice. **(H)** Representative images of infarct areas stained with picrosirius red visualized under polarized light. Scale bar: 100 μm. **(I)** Quantification of picrosirius red polarized light analysis in the infarct areas of MI mice. Dot-plots represent data from individual mice. Statistical significance was determined by a log-rank test (**C**), two-way ANOVA followed by Sidak's multiple comparisons test (**D, E, G**) or Kruskal-Wallis test followed by Dunn's posttest to correct for multiple comparisons (**G**). *P < 0.05, **P < 0.01 and ***P < 0.001 compared to the corresponding control.

To further clarify whether the monocyte/macrophage phenotypic transition was regulated by hypoxia, bone marrow cells were isolated and differentiated into macrophages (Figure 4A). The hypoxic macrophages (M_H_ macrophages) displayed a circle-like morphology with reduced pseudopodia formation (Figure 4B and Figure S13A-B). Meanwhile, hypoxia could enhance the migration ability of MH macrophages relative to MN macrophages (Figure S13C). In the analysis of M_H_ macrophages in a wide panel versus M_1_ and M_2_ macrophages, we found that the M_H_ macrophages differed from the M_1_ and M_2_ macrophages (Figure S1D-F). To trace the macrophages injected into the heart, we isolated and cultured monocytes / macrophages from CX3CR1-GFP mice (Figure S14A-B). M_H_ CX3CR1-GFP macrophages were transplanted into the myocardium immediately after left anterior descending coronary artery (LAD) ligation. The location of the CX3CR1-GFP macrophages was determined by co-immunostaining with anti-GFP and anti-F4/80 antibodies in the MI mice (Figure S14C-E). We quantified the number and identified the location (infarct, border and remote) of the transplanted CX3CR1-GFP macrophages relative to all macrophages found in different regions of the heart at various time points to further evaluate the transplantation efficacy (Figure S14F-G).

Compared with the normal macrophage transplantation, the M_H_ macrophage transplantation improved the long-term survival rate after MI (Figure 4C). The M_H_ macrophage transplantation effectively reduced ventricular remodeling and significantly decreased the heart weight-to-body weight ratio compared with those following normal macrophage transplantation (Figure 4D). Additionally, the ejection fraction (EF) and fractional shortening (FS) were preserved following the M_H_ macrophage transplantation (Figure 4E and Table S2). The LV size and morphology were also well maintained in the M_H_ macrophage-transplanted mice compared to those in the normal macrophage-transplanted mice (Figure 4F and Figure S15A). Additionally, a reduction in the scar size was observed in the M_H_ macrophage-transplanted mouse hearts (Figure 4G and Figure S15A). The tightly packed versus loosely packed fiber ratio was significantly lower in the infarcts and border zones in the M_H_ macrophage-transplanted mouse hearts (Figure 4H-I and Figure S16A-C). Moreover, fibroblast proliferation in the infarct area in the M_H_ macrophage-transplanted mouse hearts was lower than that in the other groups (Figure S16D). More capillaries and pH3-positive cardiomyocytes were observed surrounding the border zone in the MI mice transplanted with the M_H_ macrophages compared to those in the mice transplanted with normal macrophages (Figure S17A-B). These results strongly suggest that isolated monocytes/macrophages treated with hypoxia exerted a protective effect on postinfarction myocardial repair.

### Hypoxia Polarizes Unstimulated Bone Marrow-Derived Macrophages to a Pro-reparative Phenotype

**Figure 5:**
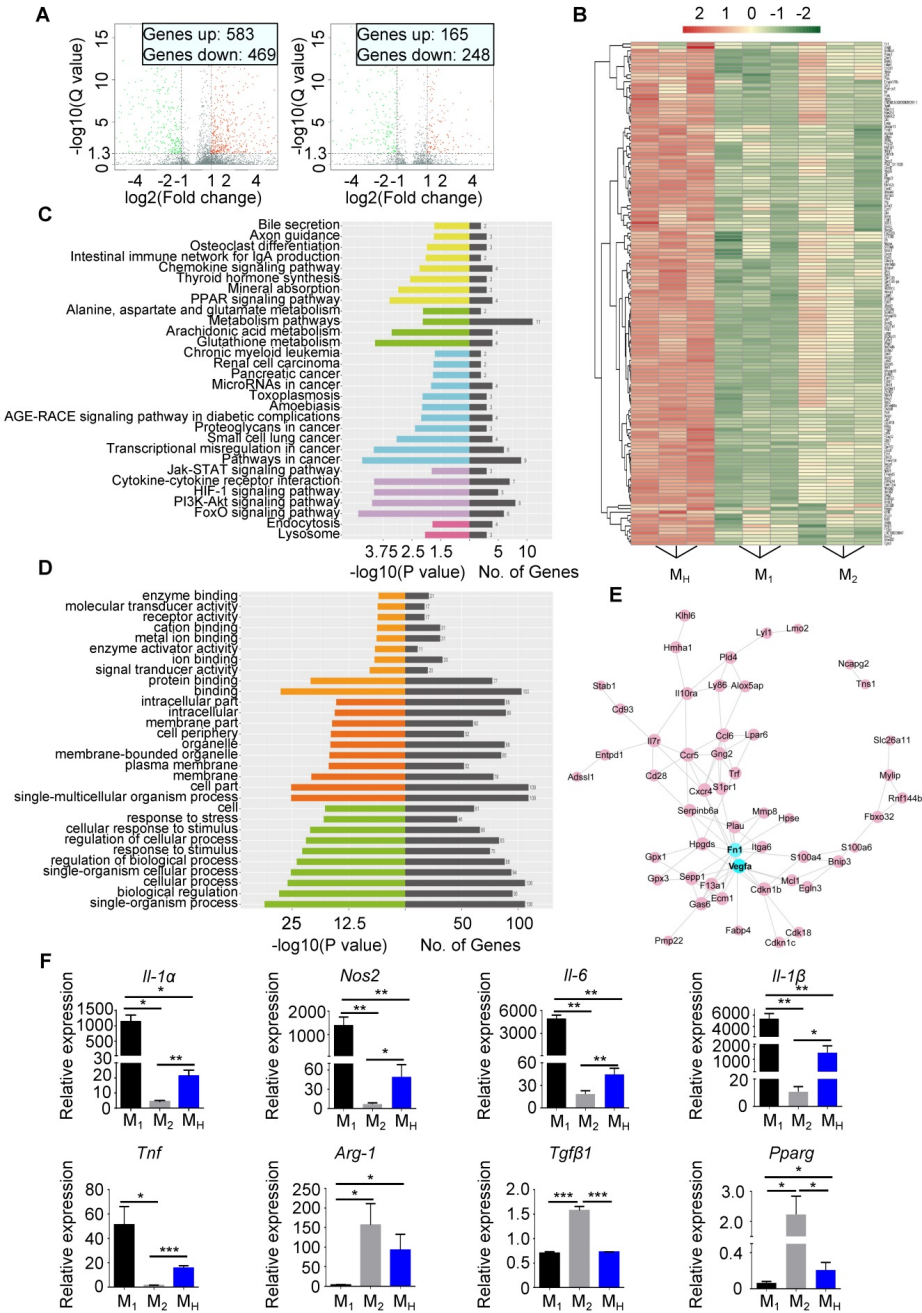
** Hypoxia polarizes isolated macrophages to a reparative phenotype. (A)** Volcano plot showing the different gene expression levels between M_1_ macrophages and M_H_ macrophages (left) or between M_2_ macrophages and M_H_ macrophages (right). **(B)** Heat map of upregulated gene expression in M_H_ macrophages relative to those in M_1_ and M_2_ macrophages. **(C)** KEGG analysis of biological pathways associated with the genes upregulated in the M_H_ macrophages relative to those in the M_1_ and M_2_ macrophages. **(D)** Functional enrichment analysis of genes upregulated in the M_H_ macrophages relative to those in the M_1_ and M_2_ macrophages. **(E)** Protein interaction network of genes upregulated in the M_H_ macrophages relative to those in the M_1_ and M_2_ macrophages. **(F)** Gene expression profiles of macrophages polarized toward the M_1_, M_2_, and M_H_ phenotypes relative to M_N_ macrophages. n = 3 per group. Statistical significance was determined by a Kruskal-Wallis test, followed by Dunn's posttest to correct for multiple comparisons. *P < 0.05, **P < 0.01 and ***P < 0.001 compared to corresponding M_N_ macrophages.

The M_H_ macrophages differed from the M_1_ and M_2_ macrophages (Figure S13A-E). A gene analysis was performed to distinguish the M_H_ macrophages from the M_1_ and M_2_ macrophages. In total, 583 genes were upregulated, while 469 genes were downregulated in the M_H_ macrophages compared to those in the M_1_ macrophages. Compared to the M_2_ macrophages, 165 genes were upregulated, and 248 genes were downregulated in the M_H_ macrophages (Figure 5A and Figure S18A-B). We filtered the genes upregulated in the M_H_ macrophages compared to the other two subtypes. The heat map shows all upregulated genes (Figure 5B). The KEGG pathway enrichment analysis and Gene Oncology analysis indicated that the upregulated genes in the M_H_ macrophages were associated with aspects of metabolism and environmental processing, such as hypoxia and nutrition depletion (Figure 5C-D). The protein interaction network analysis identified multiple functions of the M_H_ macrophages, including metabolic regulation, paracrine regulation, and especially angiogenesis as vascular endothelial growth factor (VEGF) expression was significantly upregulated (Figure 5E). Regarding the downregulated genes in the M_H_ macrophages, the protein interaction network analysis showed that CCL2, cluster of differentiation 274 (CD274, also known as programmed death-ligand 1 (PD-L1)) and interferon-induced protein 35 (IFI35) might be the core factors (Figure S18C-E).

The gene expression of M_N_ macrophages was set as the control panel. The expression levels of nitric oxide synthase 2 (Nos2), interleukin-1 alpha (IL-1α), interleukin-1β (IL-1β), interleukin-6 (IL-6) and tumor necrosis factor (TNF) in the M_H_ macrophages were all lower than those in the M_1_ macrophages but higher than those in the M_2_ macrophages (Figure 5F). Meanwhile, arginase 1 (Arg-1), peroxisome proliferator-activated receptor gamma (Pparg) and transforming growth factor beta 1 (Tgfβ1) were also changed in the M_H_ macrophages, and their expression levels were higher than those in the M_1_ macrophages but lower than those in the M_2_ macrophages (Figure 5F). Importantly, the M_H_ macrophages expressed higher levels of IL-10, which is associated with anti-inflammation, than the M_1_ and M_2_ macrophages 21 (Figure S18F). Thus, these results indicate that hypoxia-primed macrophages transform into a phenotype that prone to promoting tissue repair, and this phenotype differs from that of M_1_ and M_2_ macrophages.

### Macrophages Were Modulated by Hypoxia through the AMPKα2 Signaling Pathway

**Figure 6:**
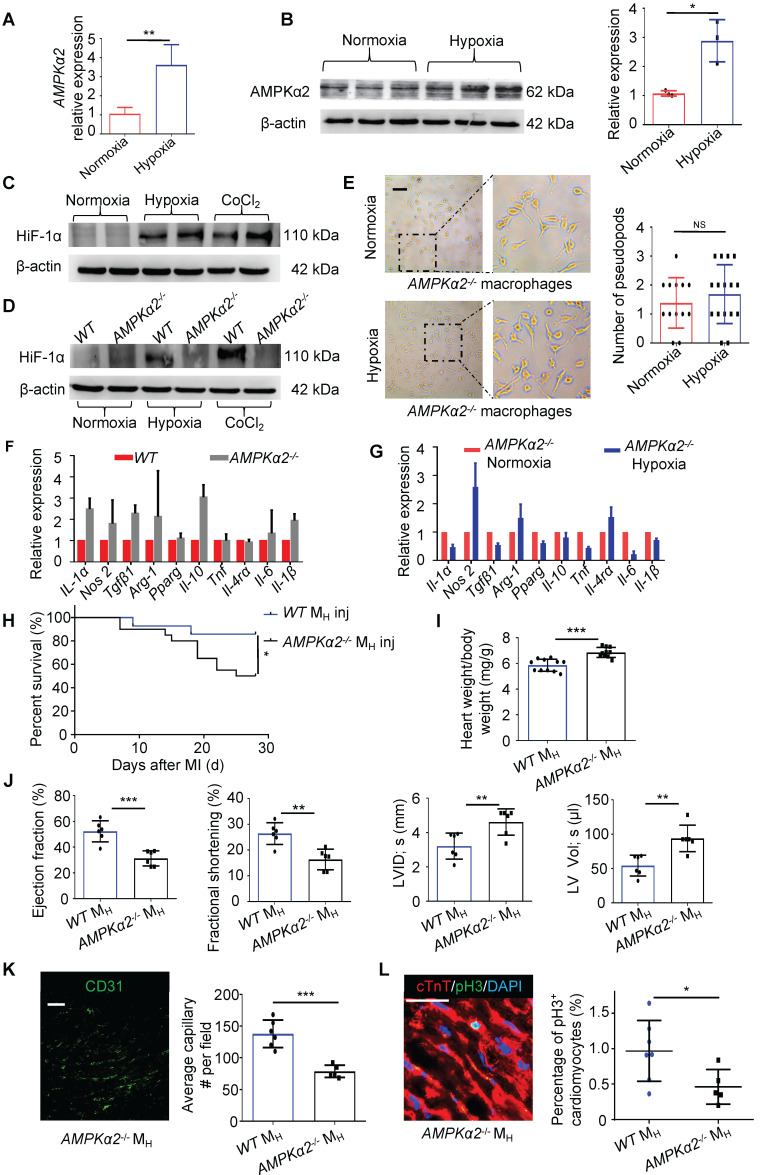
** Macrophages were polarized by hypoxia through the AMPKα2 signaling pathway. (A)** Gene expression of AMPKα2 in M_N_ macrophages and M_H_ macrophages (n = 3). **(B)** Representative immunoblots showing AMPKα2 expression and pooled data. **(C)** Representative immunoblots showing HIF-1α expression in M_N_ macrophages, M_H_ macrophages and macrophages induced by CoCl_2_. **(D)** Representative immunoblots showing HIF-1α expression in M_N_ macrophages, M_H_ macrophages and CoCl_2_-induced macrophages from wild-type (*WT*) or *AMPKα2*-knockout (*AMPKα2*^-/-^) mice. **(E)** Representative phase-contrast images of *AMPKα2*^-/-^ macrophages and pooled data showing the number of pseudopods in macrophages under normoxic and hypoxic conditions. Scale bar: 275 μm. **(F)** Gene expression profiles of normoxic macrophages from *WT* and *AMPKα2*^-/-^ mice (n = 3). **(G)** Gene expression profiles of normoxic or hypoxic *AMPKα2*^-/-^ macrophages (n = 3). **(H)** Cumulative survival curve (Kaplan-Meier survival plot) of MI mice following *WT* and *AMPKα2*^-/-^ M_H_ macrophage transplantation (n = 14 for MI followed by *WT* M_H_ macrophage transfer, n = 20 for MI followed by *AMPKα2*^-/-^ M_H_ macrophage transfer). **(I)** Heart weight-to-body weight (HW/BW) ratio after MI. **(J)** LVEF, LVFS, LVID, s and LV Vol; s were measured by echocardiography and are presented. **(K)** The ratio of anti-CD31-positive cells to anti-cTnT-positive cells was significantly decreased in the MI mice following the *AMPKα2*^-/-^ macrophage transfer. Scale bar: 50 μm. **(L)** Coimmunostaining with anti-pH3 and anti-cTnT antibodies showed significantly decreased cardiomyocyte mitosis in the MI mice following the *AMPKα2*^-/-^ M_H_ macrophage transfer. Scale bar: 50 μm. Dot-plots represent data from individual mice. Statistical significance was determined by a Mann-Whitney test (**A, B, F, G**), unpaired* t*-test (**E, I, J, K, L**) or log-rank test (**H**). *P < 0.05, **P < 0.01 and ***P < 0.001 compared to the corresponding *WT* control.

As metabolism- and stress-responsive genes were upregulated in the M_H_ macrophages, we investigated whether AMPK participates in the development of hypoxia-primed macrophages. AMPKα1, which is a key regulator of macrophage skewing during skeletal muscle regeneration 22, was not changed in the M_H_ macrophages; however, AMPKα2 was significantly upregulated at both the gene and protein levels in the macrophages (Figure 6A-C and Figure S19A-B). Thus, we postulated that the macrophages primed by hypoxia might be regulated through AMPKα2. As AMPKα2 has transcriptional regulation activity and is one of the most important regulators of hypoxia inducible factor 1α (HIF-1α) 16, we measured the protein level of HIF-1α in the M_H_ macrophages. We found that the protein levels of HIF-1α following both the hypoxia exposure and CoCl_2_ treatment, which mimics hypoxia, were hardly upregulated when AMPKα2 was abrogated (Figure 6D). Then, we isolated bone marrow cells from AMPKα2^-/-^ mice and cultured and induced these cells to differentiate into M_H_ macrophages. We found that AMPKα2^-/-^ BMDMs exposed to hypoxia still exhibited more pseudopodia formation than normoxic cells (Figure 6E), which differed from the effect on wild-type (WT) BMDMs under hypoxia (Figure 4B). No significant changes in the gene expression profiles of general M_1_ and M_2_ macrophage markers were observed in the WT and AMPKα2^-/-^ BMDMs (Figure 6F). Additionally, the AMPKα2^-/-^ BMDMs showed no obvious phenotypic transition after exposure to hypoxia relative to normoxia (Figure 6G). Additionally, the WT BMDMs showed obvious phenotypic transition after exposure to hypoxia relative to the AMPKα2^-/-^ BMDMs (Figure S20A).

After transplanting hypoxia-primed AMPKα2^-/-^ BMDMs into the myocardium after MI, we observed that after MI, the mice did not benefit from transplantation within 28 days, and the long-term survival rate did not significantly differ from that of the vehicle-transplanted mice, which was much lower than that in the hypoxia-primed WT BMDM transplantation group (Figure 6H). LV remodeling, which is indirectly expressed by the ratio of heart weight to body weight, was obvious (Figure 6I), and cardiac function remained low (Figure 6J). Angiogenesis surrounding the infarct area was crippled due to the AMPKα2^-/-^ BMDM transplantation (Figure 6K and Figure S21A). The number of pH3-positive cardiomyocytes in the AMPKα2^-/-^ hypoxic BMDM-treated mice was lower than that in the WT BMDM-treated mice (Figure 6L).

## Discussion

Since Steele* et al* first demonstrated that oxygen can alleviate angina pectoris a century ago, oxygen supplementation has become the standard treatment for patients with AMI. It appears quite logical and biologically plausible to give oxygen to patients with AMI to improve oxygenation in ischemic myocardial tissue and decrease ischemic pain. However, several clinical trials have confirmed that oxygen supplementation might be harmful or at least not beneficial for short-term or long-term mortality and increase the rehospitalization rate 8-10. Therefore, supplemental oxygen is not recommended for routine use in AMI patients without hypoxemia. However, without considering the possible systemic harmful effect of hypoxemia, whether hypoxemia is beneficial for heart recovery after AMI is unclear. In the present study, we found that systemic hypoxia could reduce the scar size, promote cardiomyocyte proliferation, and most importantly, promote heart function recovery and increase survival. Importantly, mild systemic hypoxia was tolerable in the mice after MI. To the best of our knowledge, this report is the first to show that mild hypoxia during the early period after AMI could promote myocardial infarct repair.

Studies have confirmed the close relationship between hypoxia and cardiomyocyte proliferation. The transition to an oxygen-rich postnatal environment is a key factor resulting in the cell cycle arrest of cardiomyocytes 11, which is consistent with the fact that neonatal mice gradually lose cardiomyocyte proliferation capacity in the first postnatal week 12. In adult mice, when treated with a extremely low concentration of oxygen (7% O_2_) for approximately 2 weeks, cardiomyocyte regains proliferative abilities and promotes myocardial repair through cardiomyocyte proliferation 13. These findings provide an interesting therapeutic strategy for AMI by promoting cardiomyocyte proliferation under hypoxia. In the current study, we found evidence of cardiomyocyte proliferation, such as H3 phosphorylation and positive staining for Aurora B kinase, in cardiomyocytes under hypoxia.

Myocyte metabolism is a critical factor related to the oxygen supply and is intimately linked to the lack of oxygen supply promoting preferential glucose utilization over fatty acids as a more efficient supply of ATP. Adaptive metabolism promotes fetal gene expression, including GLUT1, to prioritize glucose utilization. A previous study showed that an increase in glucose metabolism promotes cardiac regeneration in the neonatal mouse heart 23. Neonatal mice have been shown to regenerate their hearts during a transient window of time of approximately 1 week after birth 24. A previous study showed that the hypoxic nature of the zebrafish external and circulatory environments prevents the activation of the DNA damage response and cell cycle arrest of myocytes. Mitochondrial ROS-mediated activation of the DNA damage response is an important upstream event that mediates cell cycle arrest in postnatal cardiomyocytes 12. The maintenance of the regenerative capacity may be related to hypoxic conditions. We previously found that hypoxia could prolong the cardiomyocyte regeneration window in neonatal mice 25. Meanwhile, cardiomyocyte proliferation in the cyanotic infant group was also observed to be significantly increased compared with that in the acyanotic infant group 13. Furthermore, decreased mitochondrial content with corresponding changes in metabolites, decreased radical oxygen species (ROS), and decreased DNA damage were observed in hypoxic hearts 26. Hypoxia can induce a switch in energy metabolism from mitochondrial oxidative phosphorylation to glycolysis 27. These results suggest that cardiomyocyte metabolism is hindered under hypoxia, which might be another contributor to the attenuation of remodeling after MI.

The inflammatory responses play crucial role in myocardial ischemic injury and repair. Early after MI, tissue necrosis initiates inflammation and dynamically recruits monocytes/macrophages. Infarct myocardial healing begins approximately 4 days after MI in murine models 28. During the processes of myocardial injury and repair, monocytes/macrophages are vital in clearing necrotic tissue and tissue healing 29. Any treatment that impairs or deletes macrophages will damage the process of myocardial repair 30. Modulating the function of monocytes/macrophages in different ways has obtained complex results 31,32. In nonreperfusion MI, the early period of inflammation is critical for the clearance of necrotic cells. In the present study, we found that systemic tolerable hypoxia enhanced the monocyte/macrophage transition from the pro-inflammatory to the pro-reparative subtype on approximately the 5th day after MI. Importantly, this tolerable hypoxia promoted the monocyte phenotypic transition without affecting the pro-inflammatory effect in the first 3 days after MI. Therefore, this tolerable hypoxia might be an ideal strategy to promote myocardial infarct repair.

A previous study showed that hypobaric hypoxia caused by exposure to altitude results in a significant reduction in the number of circulating peripheral dendritic cells 33. Another study reported that circulating inflammatory monocytes were increased in hypoxic mice. In hypoxic mice, the percentage and number of circulating monocytes, including pro-inflammatory Ly6C^high^ monocytes, increased at different time points after hypoxic exposure 34. In contrast, we found that under the specific context of MI, hypoxia could halt inflammation in a timely manner and promote cardiac repair. Moreover, hypoxia influences the proliferation and lineage differentiation of bone marrow hematopoietic stem cells. Whether hypoxia might have an impact on myelopoiesis in the spleen or bone marrow is worthy of attention. Myeloid cells were reported to be derived from the bone marrow, and the spleen drove a substantial increase in the number of monocytes in the blood following MI 35. Monocytes are generated through two distinct cellular pathways. The earliest monocytes arise from granulocyte-monocyte progenitors (GMPs) and monocyte-dendritic cell progenitors (MDPs). GMPs and MDPs are proposed to arise from the hierarchical model of a common myeloid progenitor (CMP) 36. In our additional experiments, we found no impact on GMPs and MDPs in either proliferation assays or absolute cell numbers. This finding might be related to the short time (7 days) of hypoxia treatment after MI, proving that relatively short period of hypoxia has few side effects on MI mice.

The numbers and phenotypes of macrophages dynamically vary across different heart diseases. According to their functions and locations, different names have been used to distinguish macrophages. The terms “M_1_ macrophage” and “M_2_ macrophage” were recommended two decades ago to classify the macrophage subtypes. These terms were based on analyses of macrophages from C57BL/6 mice, and M_1_ macrophages were induced by LPS and IFN-r via the STAT-1 pathway, while M_2_ macrophages were induced by IL-4 via the STAT-6 signaling pathway. Our observations of macrophages revealed that M_H_ macrophages were distinct from M_1_ and M_2_ macrophages. We found that M_H_ macrophages possessed improved tissue repair abilities and weak inflammatory responses. Recent research shows that oxygen is immediately reduced after injury and that the phenotypic transition of macrophages occurs simultaneously 37. We propose that hypoxia regulates the inflammatory response in the border zone of MI by altering the release of inflammatory factors, shifting from a pro-inflammatory to an anti-inflammatory environment. We also observed that hypoxia could upregulate genes related to macrophage skewing in myocardial tissue, strongly suggesting that hypoxia-regulated monocytes/macrophages might be critical for cardiac repair after MI. Importantly, we confirmed that this modulation was mainly based on blood monocytes and was not regulated by the tissue microenvironment. This finding offers a great opportunity for clinical translation as we could isolate monocytes/macrophages, stimulate them with hypoxia *in vitro* and use these hypoxia-primed monocytes/macrophages *in vivo.*

The detailed molecular mechanisms of monocyte/macrophage responses to hypoxia are still largely unknown. We found that M_H_ macrophages exhibited a “pooled” change in gene expression, mainly in genes associated with metabolic modulation and hypoxia. This finding is consistent with a previous report suggesting that metabolic modulation is critical for macrophage phenotypic transition 38. Since AMPK is a key regulator of cellular energy metabolism, ROS redox balance and the interaction between HIF-1α and AMPKα have been reported in cancer cells 39. Inhibiting the expression of AMPKα impaired the expression of HIF-1α in the nucleus under hypoxia or low glucose conditions. Studies have shown that AMPKα1 is a key molecule through which macrophages promote angiogenesis and tissue repair under normoxia and that the depletion of AMPKα1 impairs macrophage promotion of arteriogenesis 40. AMPKα1 in fibroblasts was reported to function as a master regulator of cardiac fibrosis and remodeling after MI 41. However, there have only been a few reports on the role of AMPKα2 in macrophages. In contrast to the AMPKα1 subunit, AMPKα2 can modulate gene and protein expression by translocating to the nucleus through many signaling pathways. In neutrophils, AMPKα2 regulates α-ketoglutarate generation, HIF-1α stability, and neutrophil survival, which, in turn, further facilitates myeloid cell recruitment and repair potential. In the present study, we found that AMPKα2, but not AMPKα1, was upregulated in hypoxia-primed monocytes/macrophages and that AMPKα2 was a key regulator of monocyte/macrophage phenotypic transition.

The latest results demonstrated that the production of interleukin (IL)-10 by macrophages promotes anti-inflammatory functions and prevents fibrosis after tissue damage, which could lead to new therapeutic perspectives for inflammatory diseases 42. In our study, M_H_ macrophages expressed higher levels of IL-10 than M_1_ and M_2_ macrophages. IL-10 is considered to have a cardioprotective effect on improving the LV function and diminishing pathological remodeling 43. Recombinant IL-10 could preserve cardiac function, attenuate maladaptive remodeling and improve long-term survival in mice 44. IL-10 has been reported to increase the expression of intracellular galectin-3 through the activation of STAT3 in macrophages, which is essential for osteopontin-producing reparative macrophage polarization after myocardial infarction 21. We believe that inflammatory factors, such as IL-10, may be the key mediators secreted by hypoxia-primed macrophages that exert cardioprotective effects.

Currently, promising cardioprotective strategies for MI include the combination of ischemic postconditioning and remote ischemic preconditioning 45, which aims to reduce the infarct size and attenuate adverse cardiac remodeling and progression to heart failure 45. Meanwhile, clinical trials have reported that macrophage transplantation could be safe and feasible 46, 47. These results provide the basis for future prospective randomized clinical trials and hinder the potential clinical applications and future prospects of our results.

## Conclusion

Our data suggest that hypoxia promotes the monocyte/macrophage phenotypic transition from pro-inflammatory to pro-reparative in a timely manner and promotes postinfarction myocardial repair. Hypoxia priming might be an attractive translational strategy for MI treatment by amplifying immune cells during early inflammation and subsequent resolution and repair.

## Supplementary Material

Supplementary figures and tables.

## Abbreviations

MI: myocardial infarction
BMDMs: bone marrow-derived macrophages
AMPKα2: adenosine 5'-monophosphate (AMP)-activated protein kinase α2
AMI: acute myocardial infarction
STEMI: ST-elevation myocardial infarction
HIF-1α: hypoxia-inducible factor-1α
LAD: left anterior descending artery
LV: left ventricular
RBC: red blood cell
Ly6C: lymphocyte antigen 6 complex locus C
CCR2: C-C motif chemokine receptor 2
CCL2: chemokine (C-C motif) ligand 2
CXCL7: chemokine (C-X-C motif) ligand 7
LVEF: left ventricular ejection fraction
LVID: left ventricular internal diameter
VEGF: vascular endothelial growth factor
GMPs: granulocyte-monocyte progenitors
MDPs: monocyte-dendritic cell progenitors
CMP: common myeloid progenitor

## References

[1] Nahrendorf M (2018). Myeloid cell contributions to cardiovascular health and disease. Nat Med.

[2] Swirski FK, Nahrendorf M (2013). Leukocyte behavior in atherosclerosis, myocardial infarction, and heart failure. Science.

[3] Heidt T, Courties G, Dutta P, Sager HB, Sebas M, Iwamoto Y (2014). Differential contribution of monocytes to heart macrophages in steady-state and after myocardial infarction. Circ Res.

[4] Leuschner F, Nahrendorf M (2020). Novel functions of macrophages in the heart: insights into electrical conduction, stress, and diastolic dysfunction. Eur Heart J.

[5] Cao DJ, Schiattarella GG, Villalobos E, Jiang N, May HI, Li T (2018). Cytosolic DNA sensing promotes macrophage transformation and governs myocardial ischemic injury. Circulation.

[6] Steg PG, James SK, Atar D, Badano LP, Blömstrom-Lundqvist C, Borger MA (2012). ESC Guidelines for the management of acute myocardial infarction in patients presenting with ST-segment elevation. Eur Heart J.

[7] Roffi M, Patrono C, Collet JP, Mueller C, Valgimigli M, Andreotti F (2016). 2015 ESC Guidelines for the management of acute coronary syndromes in patients presenting without persistent ST-segment elevation: Task Force for the Management of Acute Coronary Syndromes in Patients Presenting without Persistent ST-Segment Elevation of the European Society of Cardiology (ESC). Eur Heart J.

[8] Hofmann R, James SK, Jernberg T, Lindahl B, Erlinge D, Witt N (2017). Oxygen therapy in suspected acute myocardial infarction. N Engl J Med.

[9] Hofmann R, Witt N, Lagerqvist B, Jernberg T, Lindahl B, Erlinge D (2018). Oxygen therapy in ST-elevation myocardial infarction. Eur Heart J.

[10] Stub D, Smith K, Bernard S, Nehme Z, Stephenson M, Bray JE (2015). Air versus oxygen in st-segment-elevation myocardial infarction. Circulation.

[11] Porrello ER, Mahmoud AI, Simpson E, Hill JA, Richardson JA, Olson EN (2011). Transient regenerative potential of the neonatal mouse heart. Science.

[12] Puente BN, Kimura W, Muralidhar SA, Moon J, Amatruda JF, Phelps KL (2014). The oxygen-rich postnatal environment induces cardiomyocyte cell-cycle arrest through DNA damage response. Cell.

[13] Nakada Y, Canseco DC, Thet S, Abdisalaam S, Asaithamby A, Santos CX (2017). Hypoxia induces heart regeneration in adult mice. Nature.

[14] DeNardo DG, Ruffell B (2019). Macrophages as regulators of tumour immunity and immunotherapy. Nat Rev Immunol.

[15] Herzig S, Shaw RJ (2018). AMPK: guardian of metabolism and mitochondrial homeostasis. Nat Rev Mol Cell Biol.

[16] Abdel Malik R, Zippel N, Frömel T, Heidler J, Zukunft S, Walzog B (2017). AMP-activated protein kinase α2 in neutrophils regulates vascular repair via hypoxia-inducible factor-1α and a network of proteins affecting metabolism and apoptosis. Circ Res.

[17] Zhang H, Liu B, Li T, Zhu Y, Luo G, Jiang Y (2018). AMPK activation serves a critical role in mitochondria quality control via modulating mitophagy in the heart under chronic hypoxia. Int J Mol Med.

[18] Thackeray JT, Hupe HC, Wang Y, Bankstahl JP, Berding G, Ross TL (2018). Myocardial inflammation predicts remodeling and neuroinflammation after myocardial infarction. J Am Coll Cardiol.

[19] Kitamura T, Qian BZ, Soong D, Cassetta L, Noy R, Sugano G (2015). CCL2-induced chemokine cascade promotes breast cancer metastasis by enhancing retention of metastasis-associated macrophages. J Exp Med.

[20] de Couto G, Liu W, Tseliou E, Sun B, Makkar N, Kanazawa H (2015). Macrophages mediate cardioprotective cellular postconditioning in acute myocardial infarction. J Clin Invest.

[21] Shirakawa K, Endo J, Kataoka M, Katsumata Y, Yoshida N, Yamamoto T (2018). IL (Interleukin)-10-STAT3-Galectin-3 axis is essential for osteopontin-producing reparative macrophage polarization after myocardial infarction. Circulation.

[22] Mounier R, Théret M, Arnold L, Cuvellier S, Bultot L, Göransson O (2013). AMPKα1 regulates macrophage skewing at the time of resolution of inflammation during skeletal muscle regeneration. Cell Metab.

[23] Fajardo VM, Feng I, Chen BY, Perez-Ramirez CA, Shi B, Clark P (2021). GLUT1 overexpression enhances glucose metabolism and promotes neonatal heart regeneration. Sci Rep.

[24] Lam NT, Sadek HA (2018). Neonatal Heart Regeneration: Comprehensive Literature Review. Circulation.

[25] Liu B, Zhang HG, Zhu Y, Jiang YH, Luo GP, Tang FQ (2017). Cardiac resident macrophages are involved in hypoxia-induced postnatal cardiomyocyte proliferation. Mol Med Rep.

[26] Kimura W, Xiao F, Canseco DC, Muralidhar S, Thet S, Zhang HM (2015). Hypoxia fate mapping identifies cycling cardiomyocytes in the adult heart. Nature.

[27] Hayashi M, Sakata M, Takeda T, Yamamoto T, Okamoto Y, Sawada K (2004). Induction of glucose transporter 1 expression through hypoxia-inducible factor 1alpha under hypoxic conditions in trophoblast-derived cells. J Endocrinol.

[28] Ruparelia N, Godec J, Lee R, Chai JT, Dall'Armellina E, McAndrew D (2015). Acute myocardial infarction activates distinct inflammation and proliferation pathways in circulating monocytes, prior to recruitment, and identified through conserved transcriptional responses in mice and humans. Eur Heart J.

[29] Peet C, Ivetic A, Bromage DI, Shah AM (2020). Cardiac monocytes and macrophages after myocardial infarction. Cardiovasc Res.

[30] Zlatanova I, Pinto C, Bonnin P, Mathieu JRR, Bakker W, Vilar J (2019). Iron regulator hepcidin impairs macrophage-dependent cardiac repair after injury. Circulation.

[31] Courties G, Heidt T, Sebas M, Iwamoto Y, Jeon D, Truelove J (2014). *In vivo* silencing of the transcription factor IRF5 reprograms the macrophage phenotype and improves infarct healing. J Am Coll Cardiol.

[32] Jia D, Jiang H, Weng X, Wu J, Bai P, Yang W (2019). Interleukin-35 promotes macrophage survival and improves wound healing after myocardial infarction in mice. Circ Res.

[33] Rohm I, Aderhold N, Ratka J, Goebel B, Franz M, Pistulli R (2016). Hypobaric hypoxia in 3000 m altitude leads to a significant decrease in circulating plasmacytoid dendritic cells in humans. Clin Hemorheol Microcirc.

[34] Florentin J, Coppin E, Vasamsetti SB, Zhao J, Tai YY, Tang Y (2018). Inflammatory macrophage expansion in pulmonary hypertension depends upon mobilization of blood-borne monocytes. J Immunol.

[35] Sreejit G, Abdel-Latif A, Athmanathan B, Annabathula R, Dhyani A, Noothi SK (2020). Neutrophil-derived S100A8/A9 amplify granulopoiesis after myocardial infarction. Circulation.

[36] Liu Z, Gu Y, Chakarov S, Bleriot C, Kwok I, Chen X (2019). Fate mapping via ms4a3-expression history traces monocyte-derived cells. Cell.

[37] Rahat MA, Bitterman H, Lahat N (2011). Molecular mechanisms regulating macrophage response to hypoxia. Front Immunol.

[38] Lampropoulou V, Sergushichev A, Bambouskova M, Nair S, Vincent EE, Loginicheva E (2016). Itaconate links inhibition of succinate dehydrogenase with macrophage metabolic remodeling and regulation of inflammation. Cell metab.

[39] Cheng SC, Quintin J, Cramer RA, Shepardson KM, Saeed S, Kumar V (2014). mTOR- and HIF-1α-mediated aerobic glycolysis as metabolic basis for trained immunity. Science.

[40] Zhu H, Zhang M, Liu Z, Xing J, Moriasi C, Dai X (2016). AMP-activated protein kinase α1 in macrophages promotes collateral remodeling and arteriogenesis in mice *in vivo*. Arterioscler Thromb Vasc Biol.

[41] Dufeys C, Daskalopoulos EP, Castanares-Zapatero D, Conway SJ, Ginion A, Bouzin C (2021). AMPKα1 deletion in myofibroblasts exacerbates post-myocardial infarction fibrosis by a connexin 43 mechanism. Basic Res Cardiol.

[42] Hoeffel G, Debroas G, Roger A, Rossignol R, Gouilly J, Laprie C (2021). Sensory neuron-derived TAFA4 promotes macrophage tissue repair functions. Nature.

[43] Zhang S, Weinberg S, DeBerge M, Gainullina A, Schipma M, Kinchen JM (2019). Efferocytosis fuels requirements of fatty acid oxidation and the electron transport chain to polarize macrophages for tissue repair. Cell metab.

[44] Verma SK, Krishnamurthy P, Barefield D, Singh N, Gupta R, Lambers E (2012). Interleukin-10 treatment attenuates pressure overload-induced hypertrophic remodeling and improves heart function via signal transducers and activators of transcription 3-dependent inhibition of nuclear factor-κB. Circulation.

[45] Heusch G (2020). Myocardial ischaemia-reperfusion injury and cardioprotection in perspective. Nat Rev Cardiol.

[46] Hu X, Huang X, Yang Q, Wang L, Sun J, Zhan H (2015). Safety and efficacy of intracoronary hypoxia-preconditioned bone marrow mononuclear cell administration for acute myocardial infarction patients: The CHINA-AMI randomized controlled trial. Int J Cardiol.

[47] Patel AN, Henry TD, Quyyumi AA, Schaer GL, Anderson RD, Toma C (2016). Ixmyelocel-T for patients with ischaemic heart failure: a prospective randomised double-blind trial. Lancet.

