# Supplementary material for: Hypoxia-primed monocytes/macrophages enhance postinfarction myocardial repair
Source: Theranostics. 2022 Jan 1;12(1):307–23. doi: 10.7150/thno.63642 (PMC8690923; doi:10.7150/thno.63642)
Supplement: Supplementary file 1 — Supplementary figures and tables. [file thnov12p0307s1.pdf]

Supplementary Material

Hypoxia-primed monocytes/macrophages enhance postinfarction myocardial repair

Yu Zhu,[1,2] Wenjuan Yang, [1,2] Hailong Wang,[1] Fuqin Tang, [1,2] Yun Zhu,[1,2] Qiong Zhu,[3] Ruiyan Ma, [1,2] Zhao Jian,[1,2,*] and Yingbin Xiao[1,2,*]

[1]Department of Cardiovascular Surgery, the Second Affiliated Hospital, Army Medical University, Chongqing, China, 400037.

[2]Vascular Injury and Repair Laboratory, the Second Affiliated Hospital, Army Medical University Chongqing, Chongqing, China, 400037.

[3]Department of Ultrasound, the Second Affiliated Hospital, Army Medical University, Chongqing, China, 400037.

* Correspondence: Yingbin Xiao (xiaoyb@tmmu.edu.cn) or Zhao Jian (zhao.j@tmmu.edu.cn)

This file includes:

Figures S1-S21

Tables S1-S6

Supplementary Figures

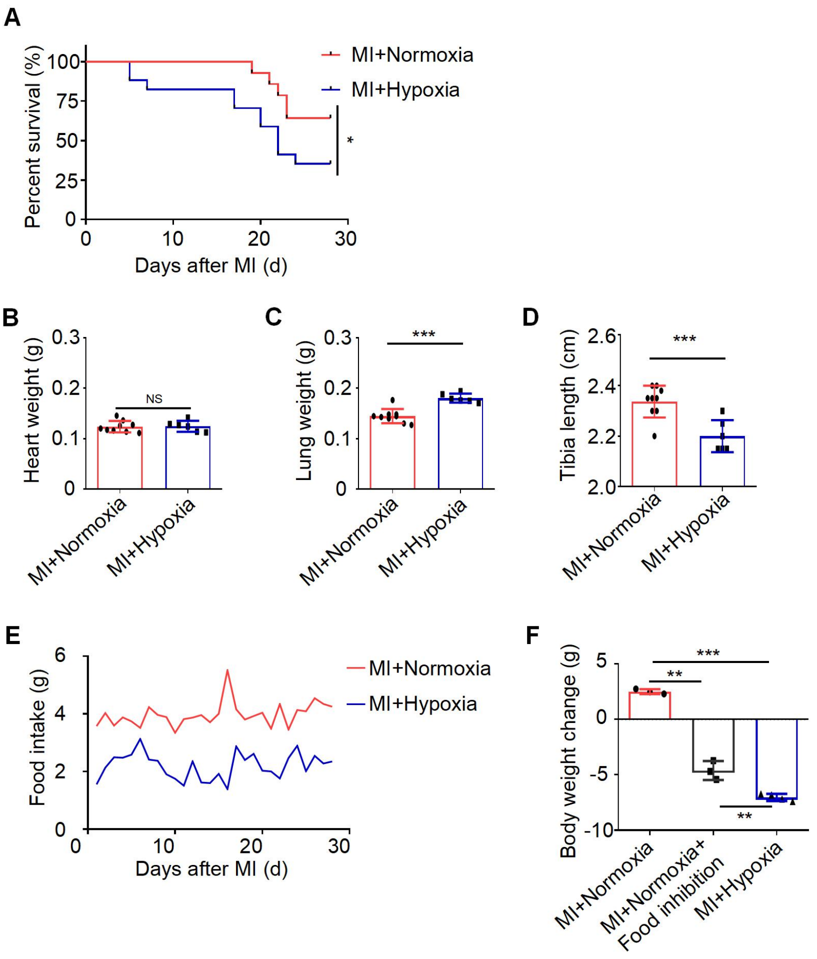

Figure S1. Long-term hypoxia has adverse effect on mice within MI. (A) Cumulative survival curve (Kaplan-Meier survival plot) of MI 28 days under hypoxia (n = 20 for normoxia MI mice and n = 19 for hypoxia MI mice). **(B)** Heart weight of MI mice 28 days under normoxia or hypoxia. **(C)** Lung weight of MI mice under 4 weeks normoxia or hypoxia. **(D)** Tibia length of MI mice 28 days under normoxia or hypoxia. **(E)** Food intake of MI mice during 28 days in normoxia or hypoxia (n = 3 for normoxia MI mice and n = 3 for hypoxia MI mice). **(F)** Body weight change of mice after 28 days within MI. Dot-plots represent data from individual mice. Statistical significance was determined by log-rank test (**A**), unpaired *t* test (**B, C, D**) or Kruskal-Wallis test, followed by a Dunn's post-test to correct for multiple comparisons (**F**). $^{NS}P > 0.05$, $*P < 0.05$, $**P < 0.01$ and $***P < 0.001$ compared to corresponding MI+normoxia.

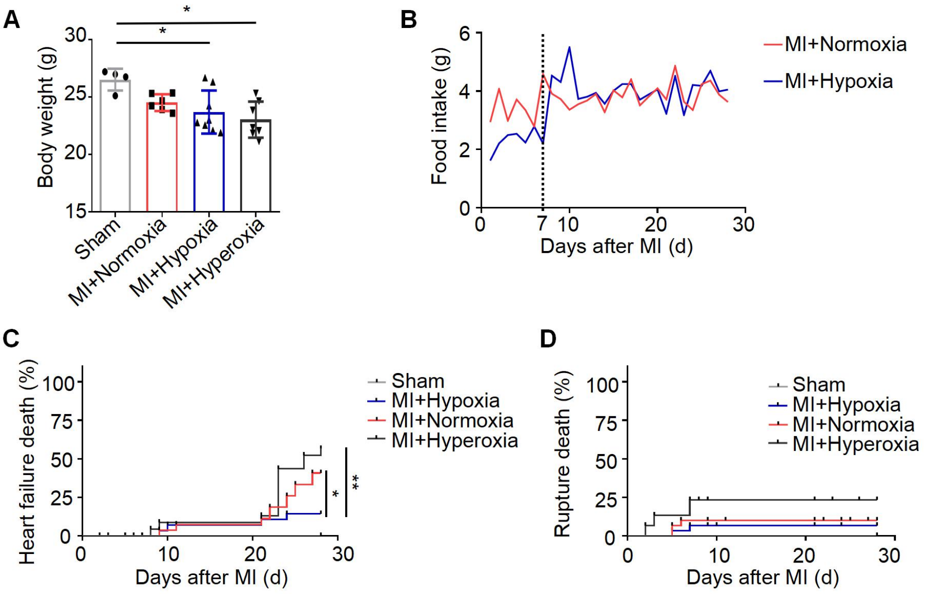

Figure S2. Short-term hypoxia after MI has no obvious adverse effect on body weight and food intake on mice. (A) Body weight of mice within MI under normoxia, hyperoxia, hypoxia and sham-operated group. **(B)** Food intake in 4 weeks of mice within MI under normoxia or hypoxia at the first week (n = 3 for each group). **(C)** Heart failure deaths of MI mice under hypoxia, normoxia and hyperoxia and sham-operated mice. **(D)** Cardiac rupture deaths of MI mice under hypoxia, normoxia and hyperoxia and sham-operated mice (n = 30 for the MI + hypoxia group, n = 30 for the MI + normoxia mice group, n = 30 for the MI + hyperoxia group, and n = 10 for the sham-operated group). Dot-plots represent data from individual mice. Statistical significance was determined by ANOVA followed by Sidak's multiple comparisons test (**A**) or log-rank test (**C**, **D**). *$P < 0.05$ compared to corresponding MI+normoxia or Sham.

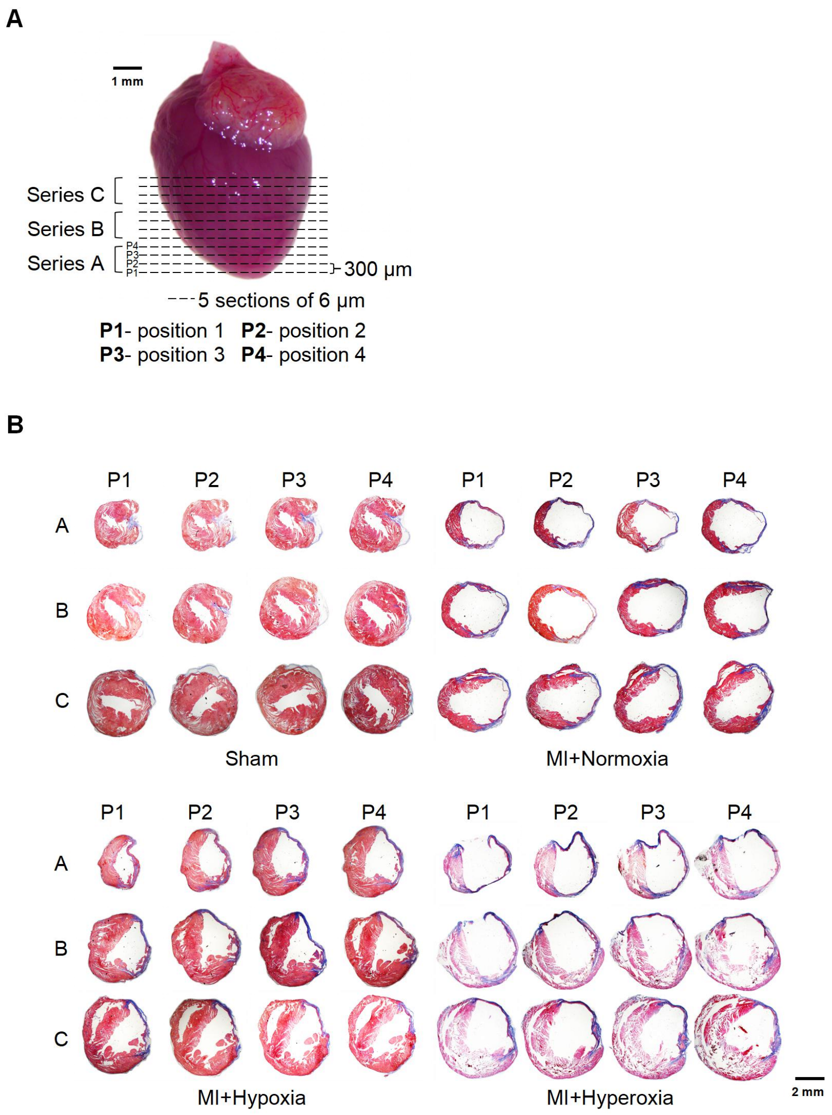

Figure S3. Overview of representative left ventricle (LV) cross sections obtained from mice heart sample with described protocol. (A) Schematic representation of the diastolic left ventricle (LV) sampling. Sectioning starts in the apical region, and five adjacent sections are collected from 12 equally distant (200-µm) regions. Scale bar: 1 mm. **(B)** Low-magnification photographs of heart sections stained with Masson's Trichrome from an infarcted mouse (LAD ligation) collected 28 days postinfarction. Series A represents the apical region of the heart, series B represents the middle zone, and series C corresponds to the base of the heart. Scale bar: 2 mm.

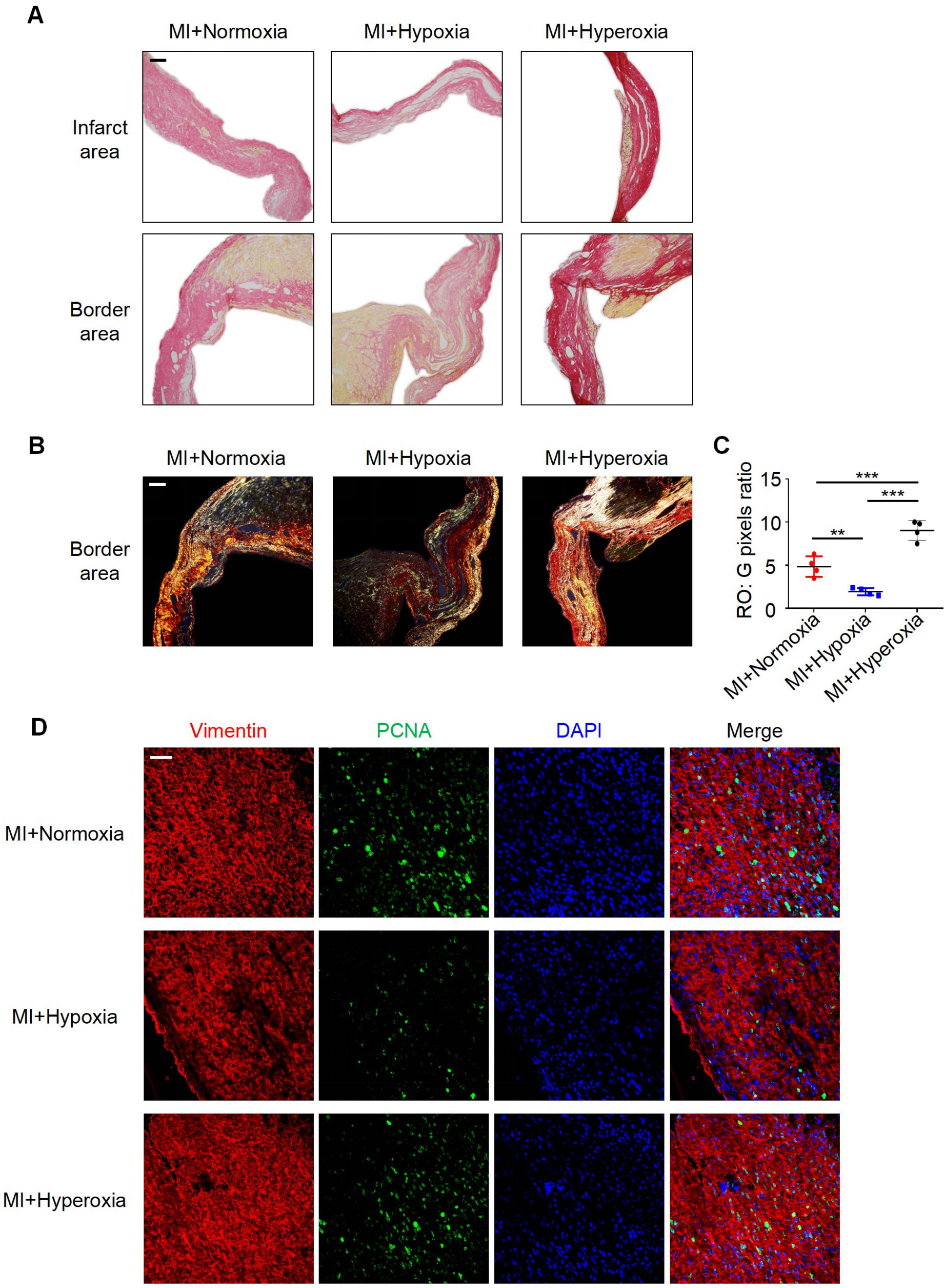

Figure S4. Hypoxia alleviated collagen deposition and LV fibrosis post-MI. (A) Representative images of infarct areas stained by picrosirius red and visualized under bright-field light. Scale bars: 100 μm. **(B)** Representative images of infarct areas stained by picrosirius red and visualized under polarised light. Scale bars: 100 μm. **(C)** Quantification of picrosirius red polarized light analysis in the infarct areas of MI mice. **(D)** Representative images of co-immunofuorescent staining of proliferating cardiac fibroblasts in the infarct area of mice under normoxia, hypoxia and hyperoxia at 7 days post-myocardial infarction (MI). Scale bars: 50 μm. Dot-plots represent data from individual mice. Statistical significance was

determined by ANOVA followed by Sidak's multiple comparisons test (**C**) or unpaired *t* test (**D**). $^{**}P < 0.01$ and $^{***}P < 0.001$ compared to corresponding control.

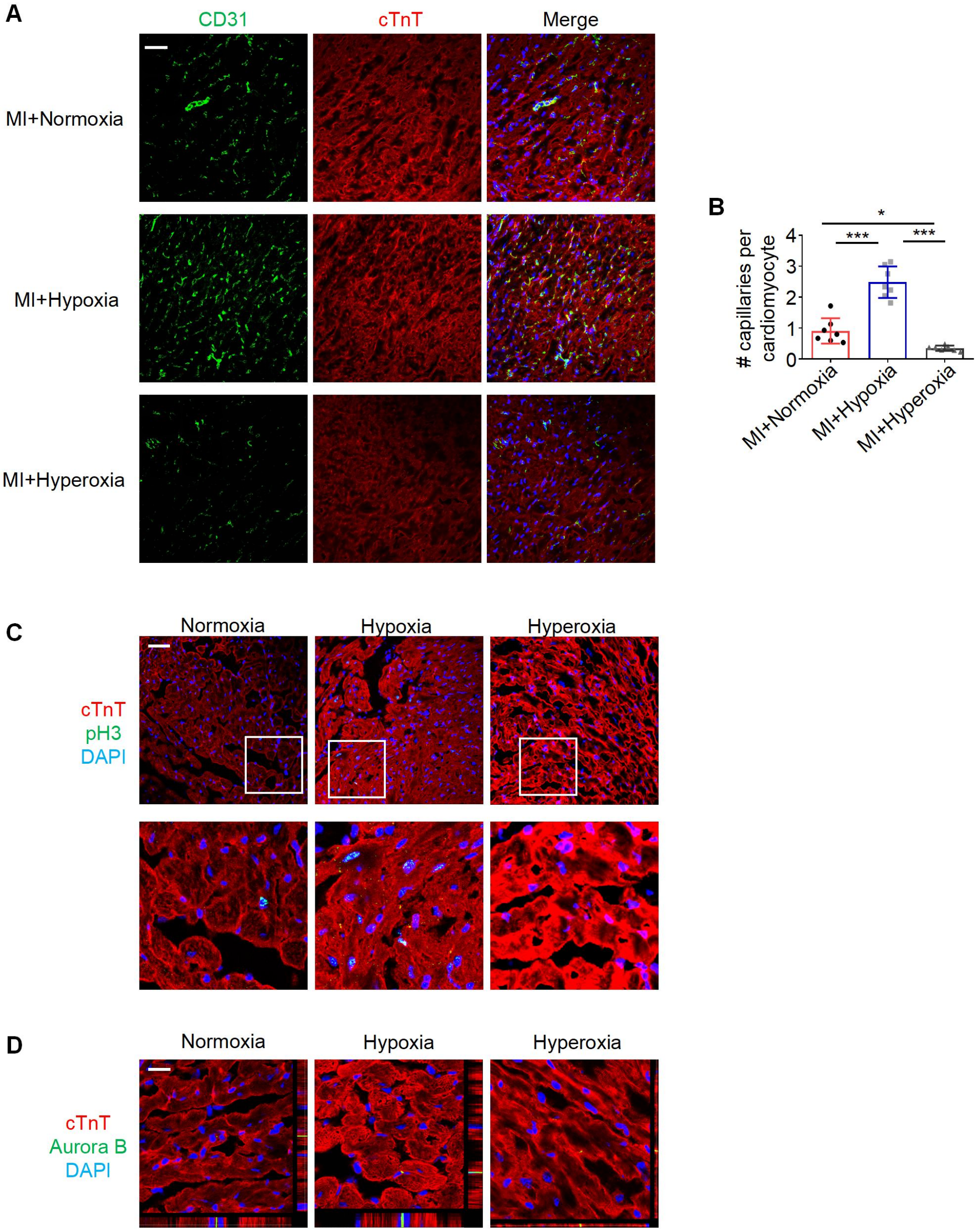

Figure S5. Hypoxia induced angiogenesis and promoted cardiomyocytes proliferation after MI. (A) Representative immunofluorescence images of CD31 stained heart tissue in border zone. Scale bar: 50 μm. **(B)** Quantification of capillaries per cardiomyocyte in border zone. **(C)** Representative immunofluorescence images and its magnified pictures of pH3 stained heart tissue in border zone. Scale bar: 50 μm. **(D)** Representative immunofluorescence images of Aurora B stained heart tissue in border zone. Scale bar: 50 μm. Dot-plots represent data from individual mice. Statistical significance was determined by ANOVA followed by

Sidak’s multiple comparisons test. *P < 0.05 and ***P < 0.001 compared to corresponding MI+normoxia.

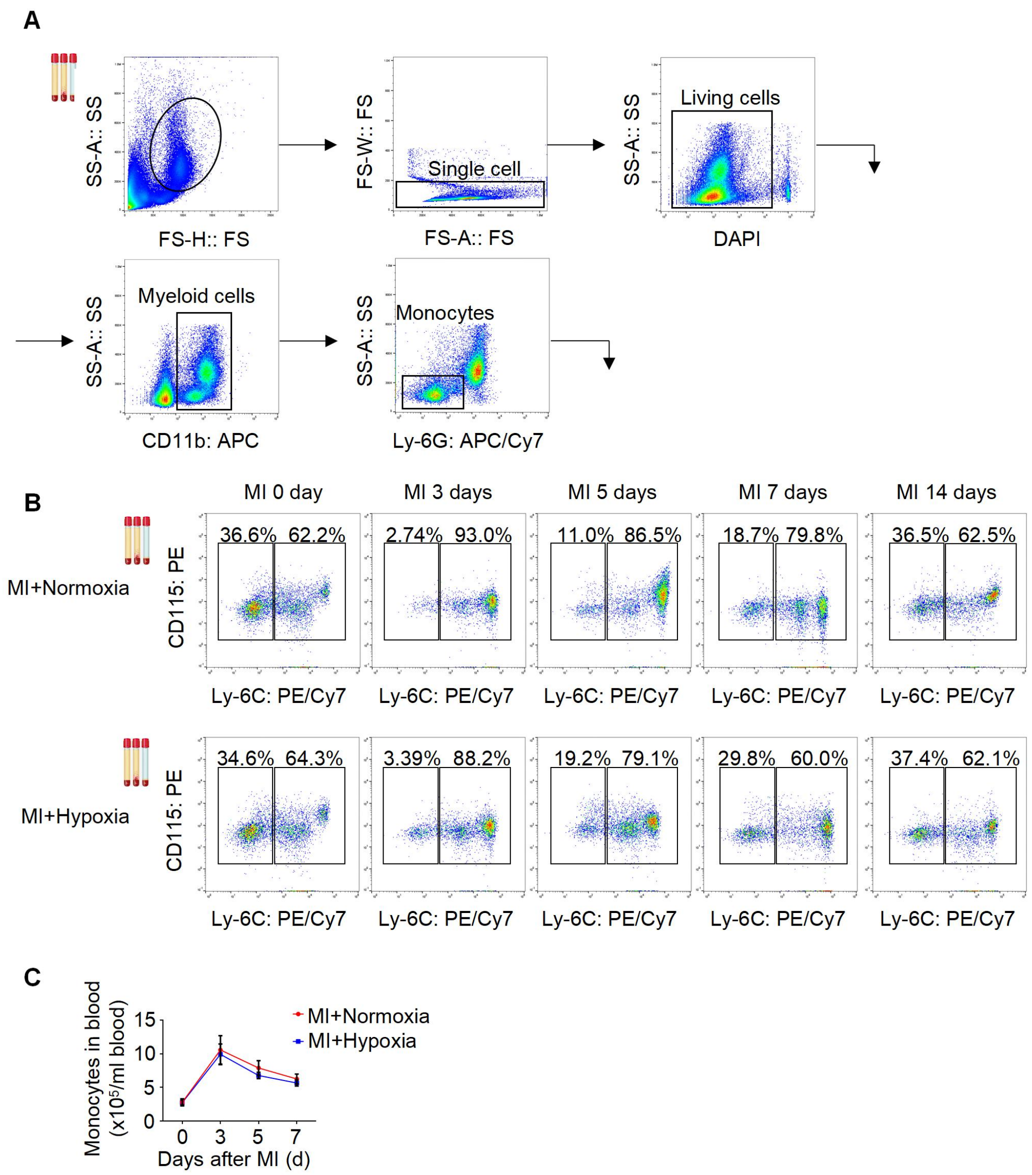

Figure S6. Systemic hypoxia promoted monocytes phenotype transition in blood after MI. (A) Gating strategy for monocyte identification in blood prior to subset analysis. Cell debris and adherent cells were excluded. Living cells were identified by DAPI^{-}. CD11b^{+} cells were identified (SSC-A/CD11b^{+}), then neutrophils were excluded (CD45^{+}/Ly-6G^{+}), monocytes in blood were divided into different subtypes by Ly-6C (CD11b^{+}/Ly-6G^{-}/CD115^{+}). **(B)** Flow cytometric plots show the proportion of Ly-6C^{high} and Ly-6C^{low} monocytes in blood samples at various time points after MI in normoxia or hypoxia group. **(C)** Quantitation of total monocytes from blood at various time points after MI in normoxia or hypoxia group (n = 3 each). Statistical significance was determined by Mann–Whitney test. $^{NS}P > 0.05$ compared to MI+Normoxia.

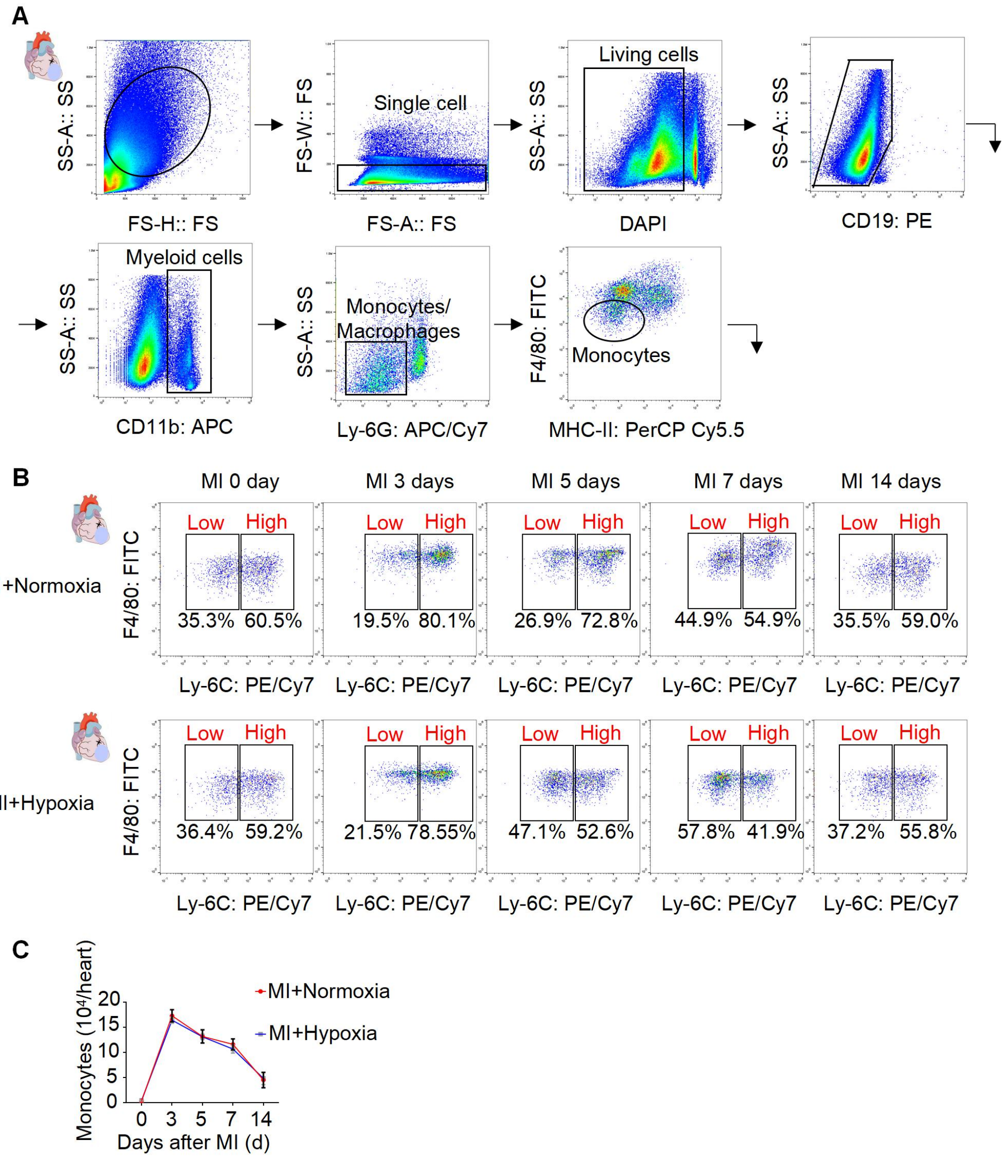

Figure S7. Systemic hypoxia promoted monocytes/macrophages phenotype transition in heart tissue after MI. (A) Gating strategy for monocyte identification in heart tissue prior to subset analysis. Cell debris and adherent cells were excluded. Living cells were identified by DAPI-. $CD19^-/CD11b^+$ cells were identified ($CD19^-/CD11b^+$), then neutrophils were excluded (Ly-$6G^+$), monocytes / macrophages in heart tissue were divided into different subtypes by Ly-6C. **(B)** Flow cytometric plots show the proportion of Ly-$6C^{high}$ and Ly-$6C^{low}$ monocytes/macrophages in heart tissue at various time points after MI in normoxia or hypoxia group. **(C)** Quantitation of total monocytes in heart tissue at various time points after

MI in normoxia or hypoxia group (n = 3 each). Statistical significance was determined by Mann–Whitney test. $^{NS}P > 0.05$ compared to MI+Normoxia.

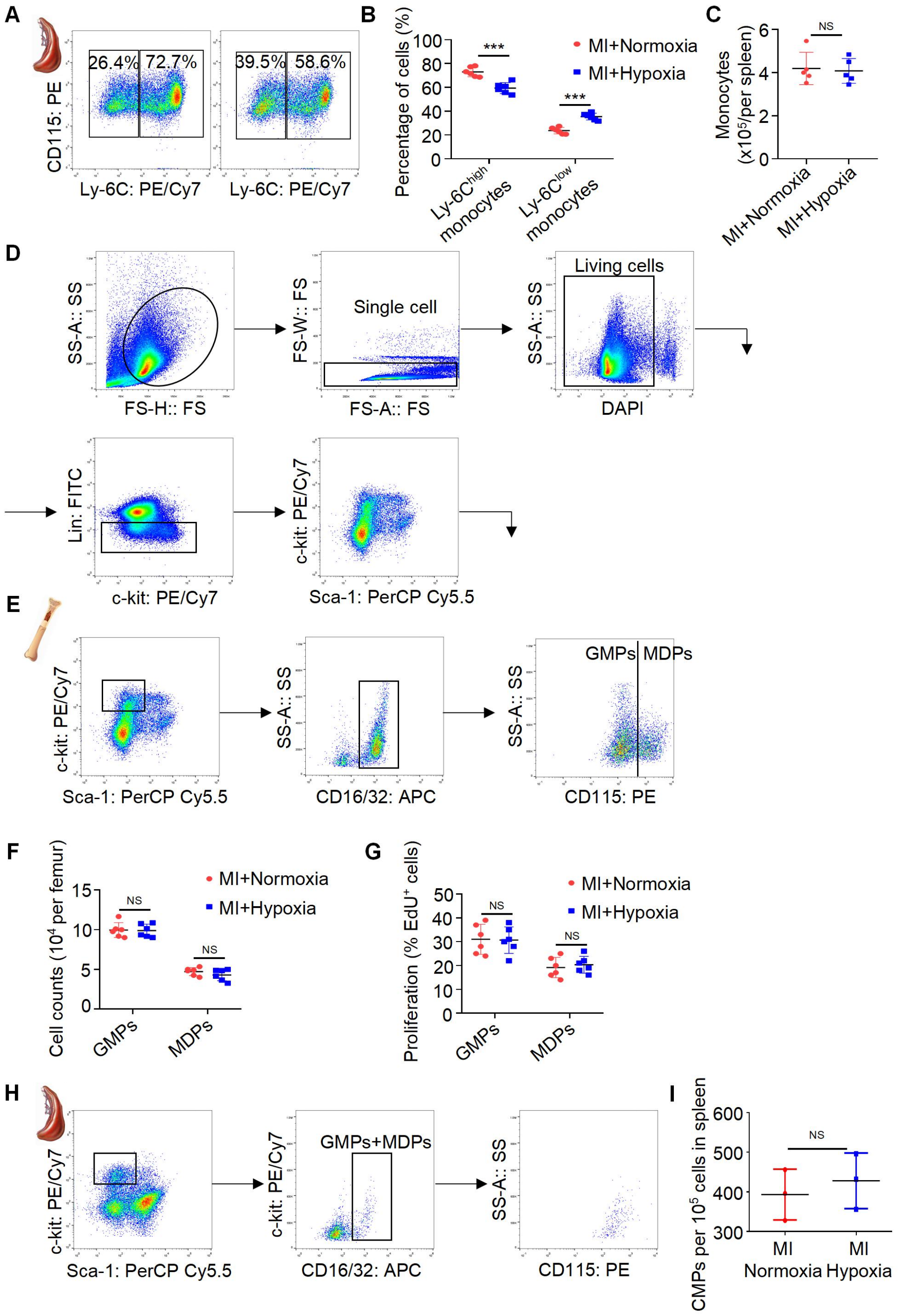

Figure S8. Systemic hypoxia had no major impact on the myelopoiesis in bone marrow and spleen. (A) Representative flow cytometric analysis of monocytes from spleen at 5 days after MI under normoxia or hypoxia. **(B)** The ratios of Ly6C^{high} and Ly6C^{low} splenic monocytes among total monocytes in spleen. **(C)** The absolute cell numbers of total splenic monocytes. **(D)** Gating strategy for myelopoiesis in the spleen and bone marrow**.** Cell debris and adherent cells were excluded. cell suspensions were labeled with FITC-conjugated

anti–mouse antibodies directed against B220, CD4, CD8a, CD11b, CD11c, Gr-1, IL7Rα, NK1.1 and Ter-119. This was followed by a second staining for CD16/32, CD115, c-Kit and Sca-1. **(E)** Common myeloid progenitor (CMPs) were defined as (B220/CD4/CD8a/CD11b/CD11c/Gr-1/IL7Rα/NK1.1/Ter-119)^{low} c-Kit^{high} Sca-1^{low} (CD16/32)^{high}, Granulocyte macrophage progenitors (GMPs) were CD115^{low} and monocyte-macrophage dendritic cell progenitors (MDPs) were CD115^{high}. **(F)** The absolute cell numbers of GMPs and MDPs in bone marrow. **(G)** Quantification of proliferation rates (assessed by EdU incorporation) of GMPs and MDPs in the BM. **(H)** Common myeloid progenitor (CMPs) in spleen were defined as (B220/CD4/CD8a /CD11b/CD11c/Gr-1/IL7Rα/NK1.1/Ter-119)^{low} c-Kit^{high} Sca-1^{low} (CD16/32)^{high}. **(I)** The numbers of CMPs among total cells in the spleen. Dot-plots represent data from individual mice. Statistical significance was determined by unpaired *t* test (**C, F, G**) or Mann–Whitney test (**I**). $^{NS}P > 0.05$ and $^{***}P < 0.001$ compared to MI+Normoxia.

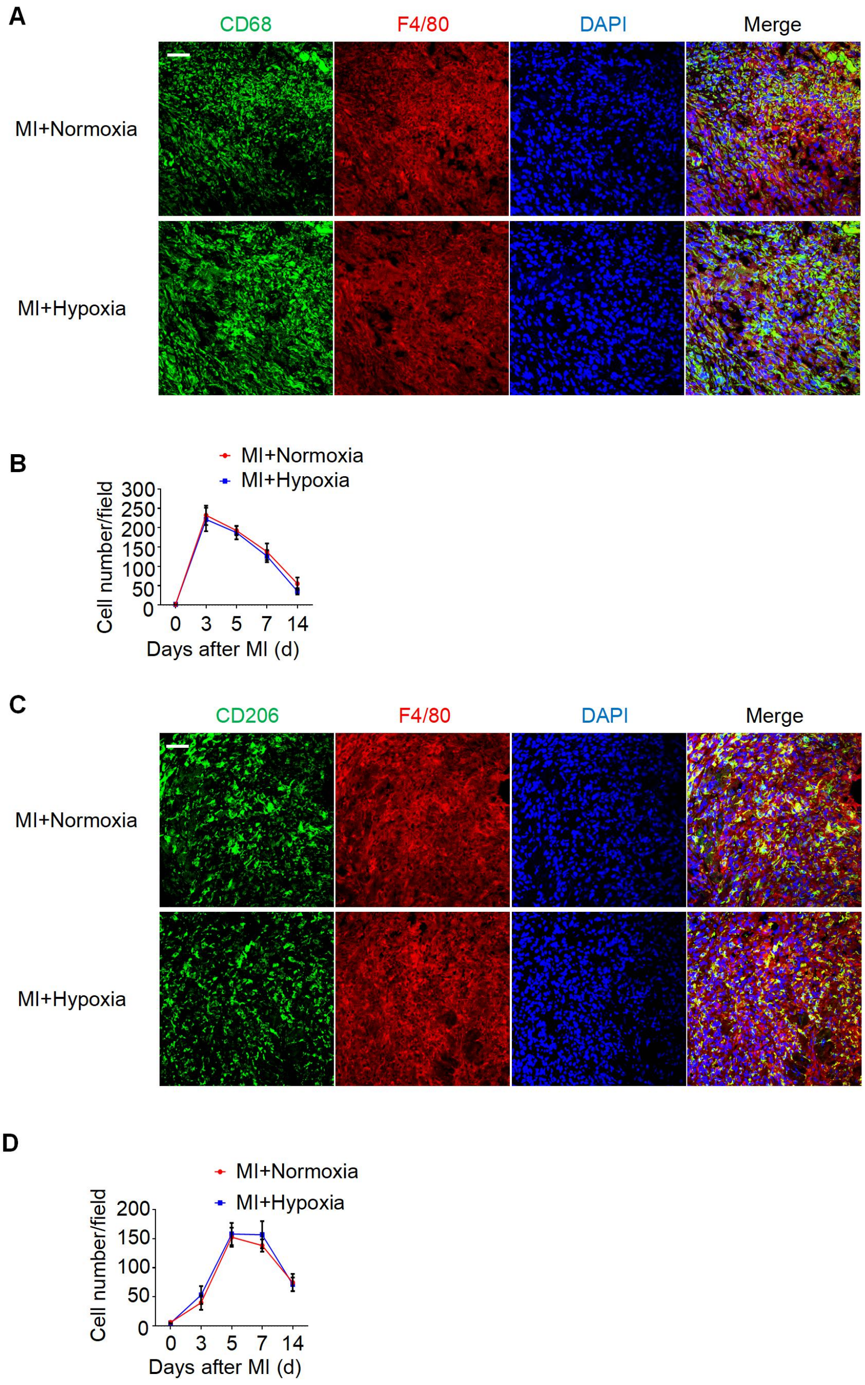

Figure S9. Hypoxia had no impact on populations of M_1/M_2 macrophages in infarct area after MI. (A) Representative immunofluorescence images of M_1 macrophages with anti-CD68 and anti-F4/80 antibodies within the infarct zone over 14 days in mice after LAD ligation. Scale bar: 50 µm. **(B)** Cell counts of M_1 macrophages in infarct area each field at various time points after MI in normoxia or hypoxia group (n = 4 each). **(C)** Representative immunofluorescence images of M_2 macrophages with anti-CD206 and anti-F4/80 antibodies within the infarct zone over 14 days in mice after LAD ligation. Scale bar: 50 µm. **(D)** Cell

counts of M_2 macrophages in infarct area each field at various time points after MI in normoxia or hypoxia group (n = 4 each). Statistical significance was determined by Mann-Whitney test (**B, D**). $^{NS}P > 0.05$ compared to corresponding MI+normoxia.

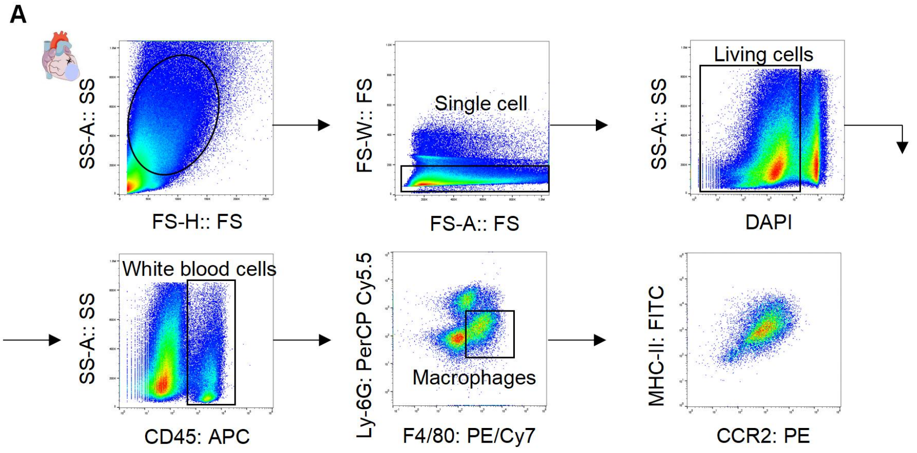

Figure S10. Monocyte-derived macrophages overwhelmed the majority in heart tissue after MI. (A) Gating strategy for monocyte-derived macrophages identification within the infarcted myocardium prior to subset analysis. Cell debris and adherent cells were excluded. Living cells were identified by $DAPI^-$. $CD45^+$ cells were first identified ($SSC\text{-}A/CD45^+$), then neutrophils were excluded ($Ly\text{-}6G^+$), macrophages were identified ($CD45^+/F4/80^+$), $CCR2^+/MHC\text{-}II^+$ macrophages are recognized monocyte-derived macrophages.

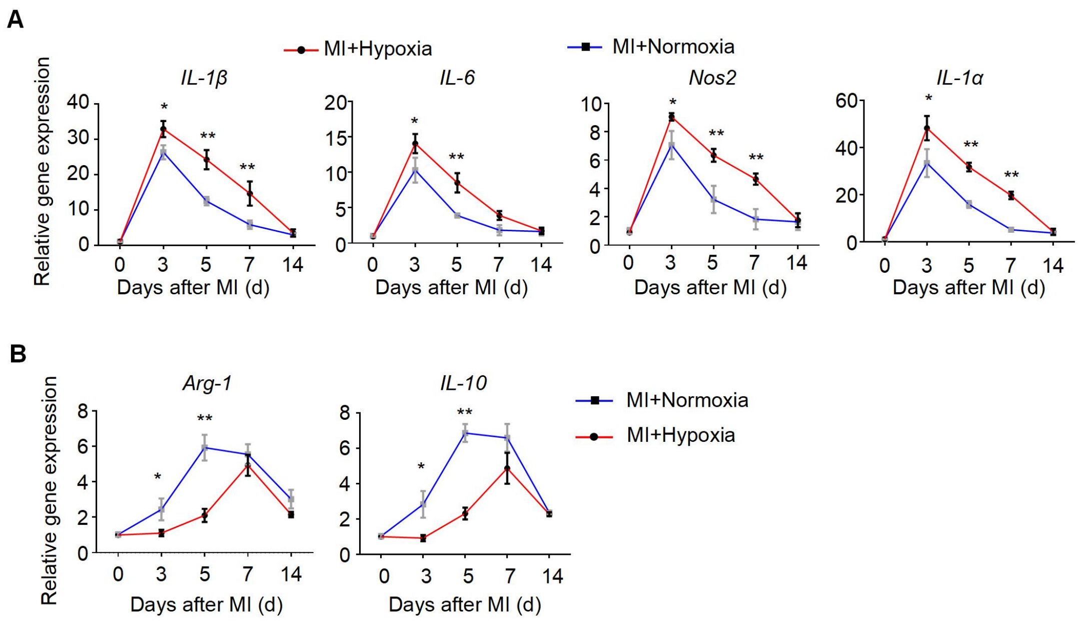

Figure S11. Hypoxia modulated the expression of cytokines associated with inflammation in the ischemic zone in the MI hearts. (A, B) Protein expression of inflammatory cytokines in the infarct zones of hearts treated over time after LAD ligation (n = 3 per group). Statistical significance was determined by a Mann-Whitney test (**A, B**) $^*P < 0.05$ and $^{**}P < 0.01$ compared to the corresponding MI+normoxia group.

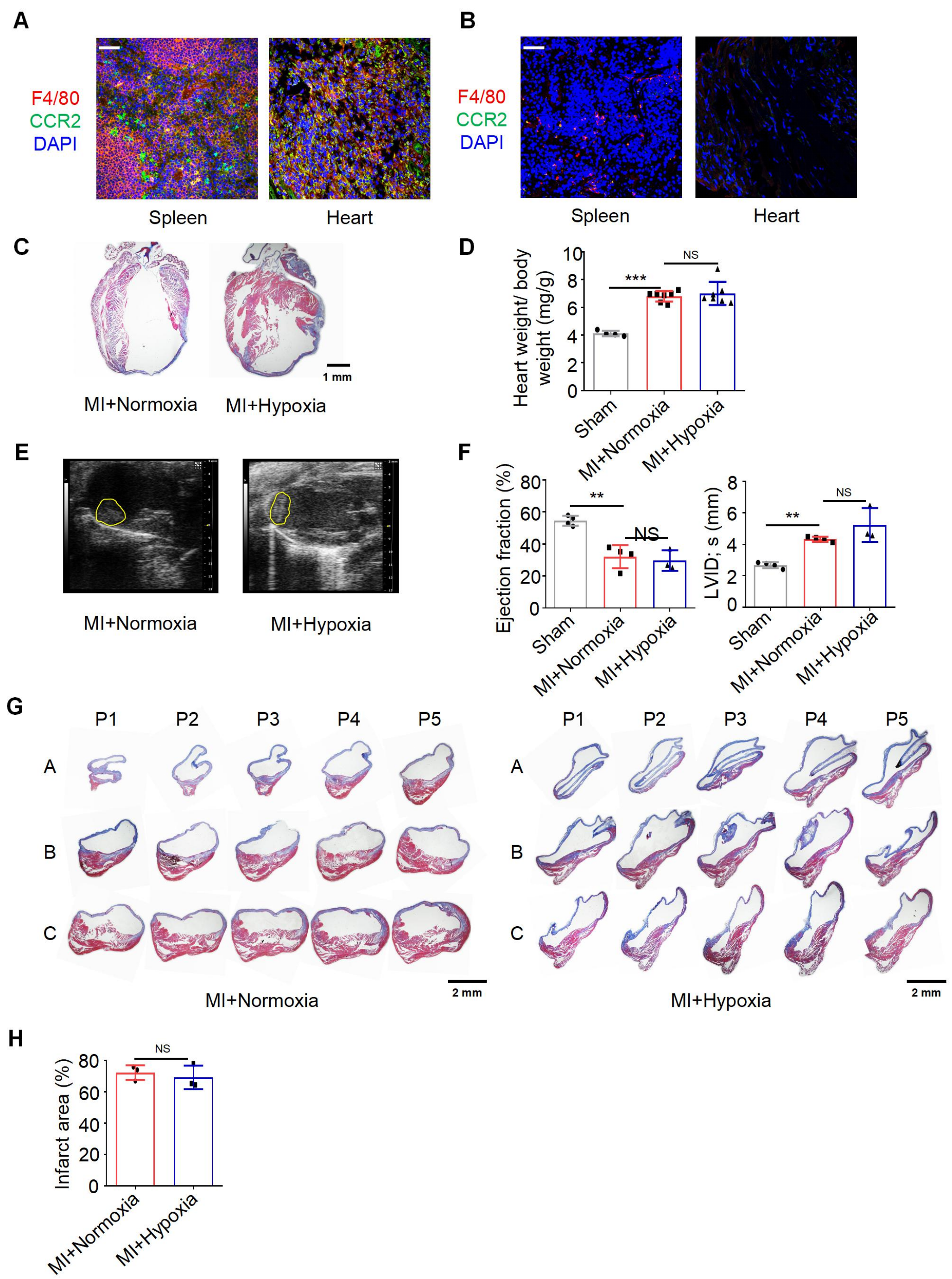

Figure S12. Systemic depletion of macrophages impaired the efficacy of hypoxia-induced cardiac protection. (A) Representative laser confocal microscope images of macrophages in spleen and heart 5 days after MI from control group. Scale bar: 50 μm. **(B)** Representative laser confocal microscope images of macrophages in spleen and heart 5 days after MI from Cl_2MDP-treated group. Scale bar: 50 μm. **(C)** Representative results of trichrome staining of longitudinal heart sections from MI mice treated with Cl_2MDP. Scale bar: 1 mm. **(D)** Heart-weight to body weight (HW/BW) ratio in sham, MI + normoxia and MI

+ hypoxia groups treated with Cl_2MDP. **(E)** Left ventricular aneurysm and mural thrombus were detected by echocardiography (n = 3 at least). **(F)** Left ventricular ejection fraction (LVEF), left ventricular internal diameter at end-systole (LVID, s) were measured using echocardiography and are presented. **(G)** Low-magnification photographs of heart sections stained with Masson's Trichrome from an infarcted mouse (LAD ligation) collected 28 days postinjury. Series A represents the apical region of the heart, series B represents the middle zone, and series C corresponds to the base of the heart. Scale bar: 2 mm. **(H)** Quantification of the fibrotic area relative to the myocardium in transverse sections. Dot-plots represent data from individual mice. Statistical significance was determined by two-way ANOVA followed by Sidak's multiple comparisons test (**E**), Kruskal-Wallis test, followed by a Dunn's post-test to correct for multiple comparisons (**F**) or Mann–Whitney test (**H**). $^{NS}P > 0.05$, $^{**}P < 0.01$ and $^{***}P < 0.001$ compared to corresponding control.

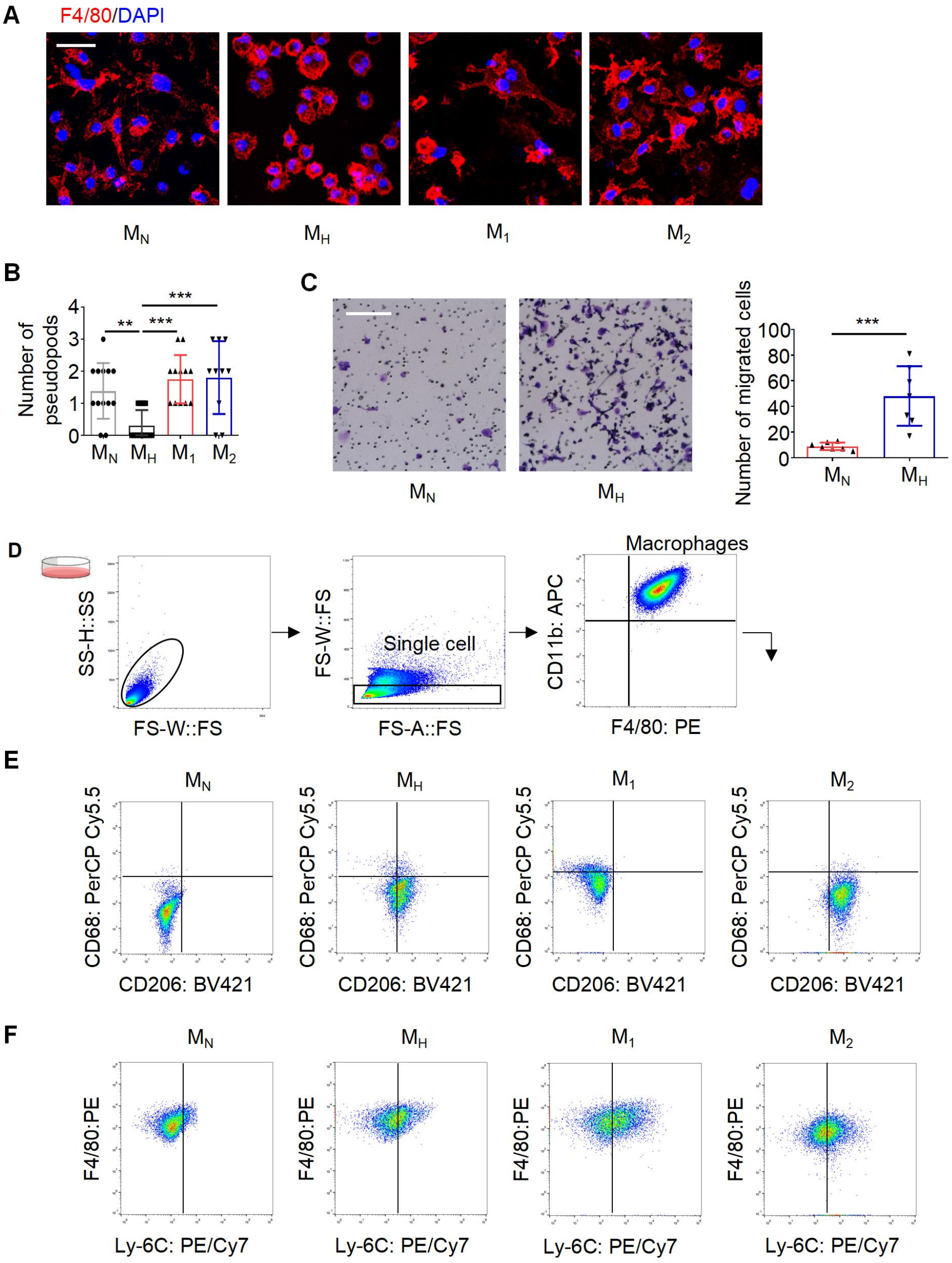

Figure S13. Comparison of M_H macrophages relative to M_1 and M_2 macrophages. (A) Representative laser confocal microscope images of macrophages polarized toward M_N, M_H, M_1 and M_2 phenotypes. Scale bar: 50 µm. **(B)** Pooled analyses of pseudopods and relative area of different types of macrophages. **(C)** The migration capacity of macrophages under hypoxia was determined using Transwell assays. Scale bar: 50 µm. **(D)** Gating strategy for bone marrow derived macrophages prior to subset analysis. BMDMs were first gated to remove debris and conjugates. Mature BMDMs were defined as CD11b$^+$F4/80$^+$ subpopulations (upper right). **(E, F)** A wide panel of M_H macrophages versus M_1 and M_2 macrophages. Dot-plots

represent data from individual mice. Statistical significance was determined by Kruskal-Wallis test, followed by a Dunn's post-test to correct for multiple comparisons (**B**) and unpaired *t* test (**C**). $^{*}P < 0.05$, $^{**}P < 0.01$ and $^{***}P < 0.001$ compared to M_N macrophages.

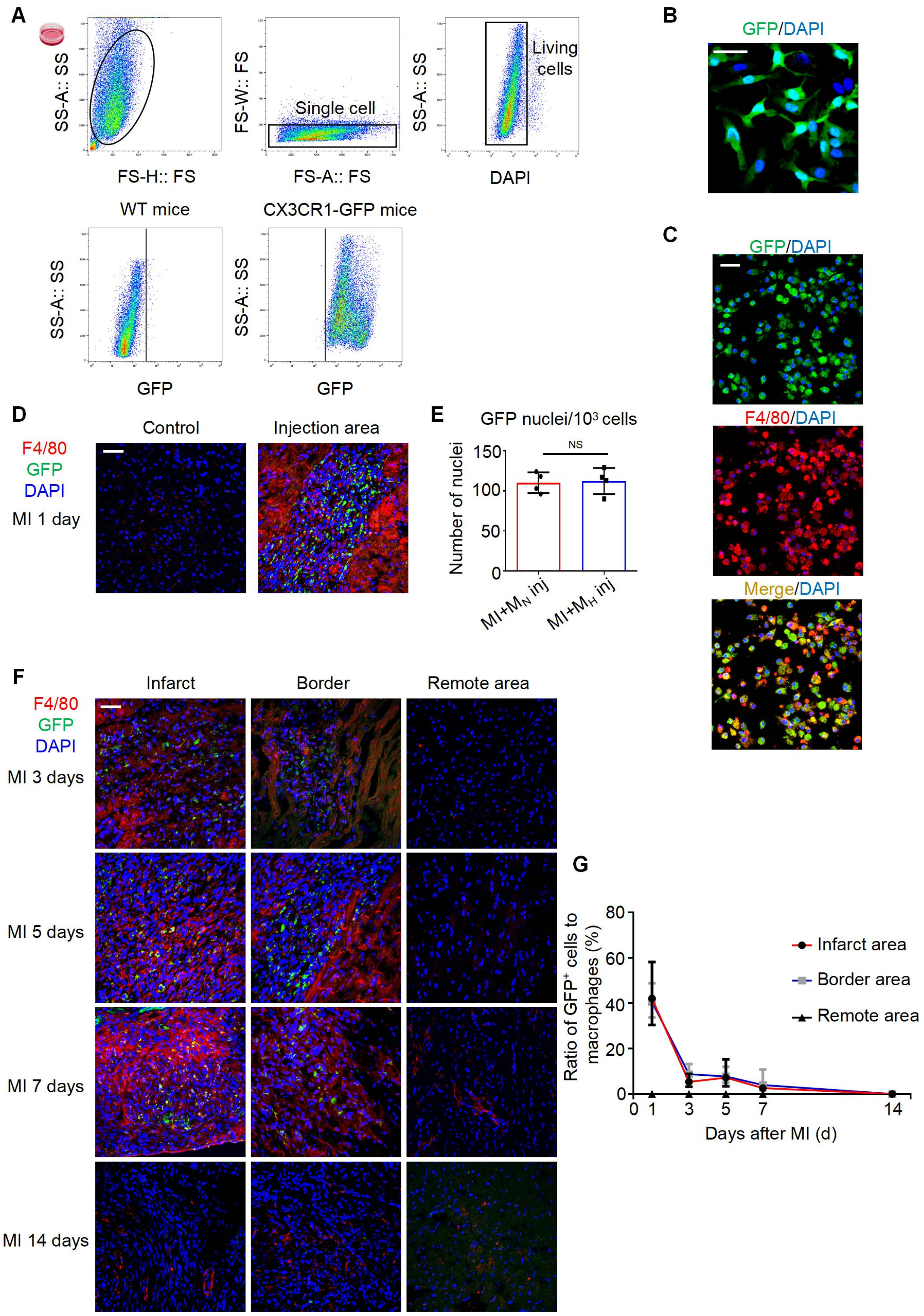

Figure S14. Evaluation of macrophages transplantation efficacy. (A) Gating strategy for cultured CX3CR1-GFP macrophages prior to analysis. Scale bar: 50 μm. **(B)** Representative immunofluorescence images of cultured CX3CR1-GFP macrophages. **(C)** Representative co-immunostaining images of CX3CR1-GFP and anti-F4/80 in cultured macrophages before

transplantation. Scale bar: 50 μm. **(D)** Representative co-immunostaining images of CX3CR1-GFP and anti-F4/80 in heart tissue following macrophages injection. Scale bar: 50 μm. **(E)** Quantification of GFP macrophages in the myocardium on first day post-MI. **(F)** Representative co-immunostaining images of CX3CR1-GFP and anti-F4/80 in different location of the heart at various time after MI. Scale bar: 50 μm. **(G)** Quantity ratio of GFP cells to macrophages in MI mouse hearts in different locations at various time. (n= 3 each). Dot-plots represent data from individual mice. Statistical significance was determined by Mann–Whitney test (e, g). $^{NS}P > 0.05$ compared to control.

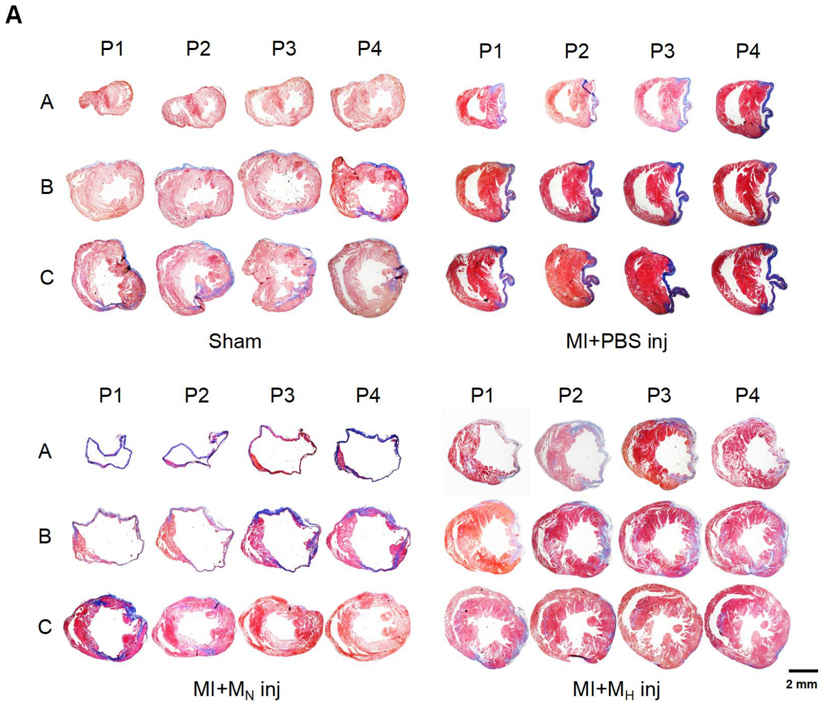

Figure S15. Overview of representative infarct area obtained from MI mice with macrophages transfer. (A) Overview of representative left ventricle (LV) cross-sections obtained from MI mice heart sample with macrophages transferred. Low-magnification photographs of heart sections stained with Masson's Trichrome from an infarcted mouse (LAD ligation) collected 28 days post-injury. Series A represents the apical region of the heart, series B represents the middle zone, and series C corresponds to the base of the heart. Scale bar: 2 mm.

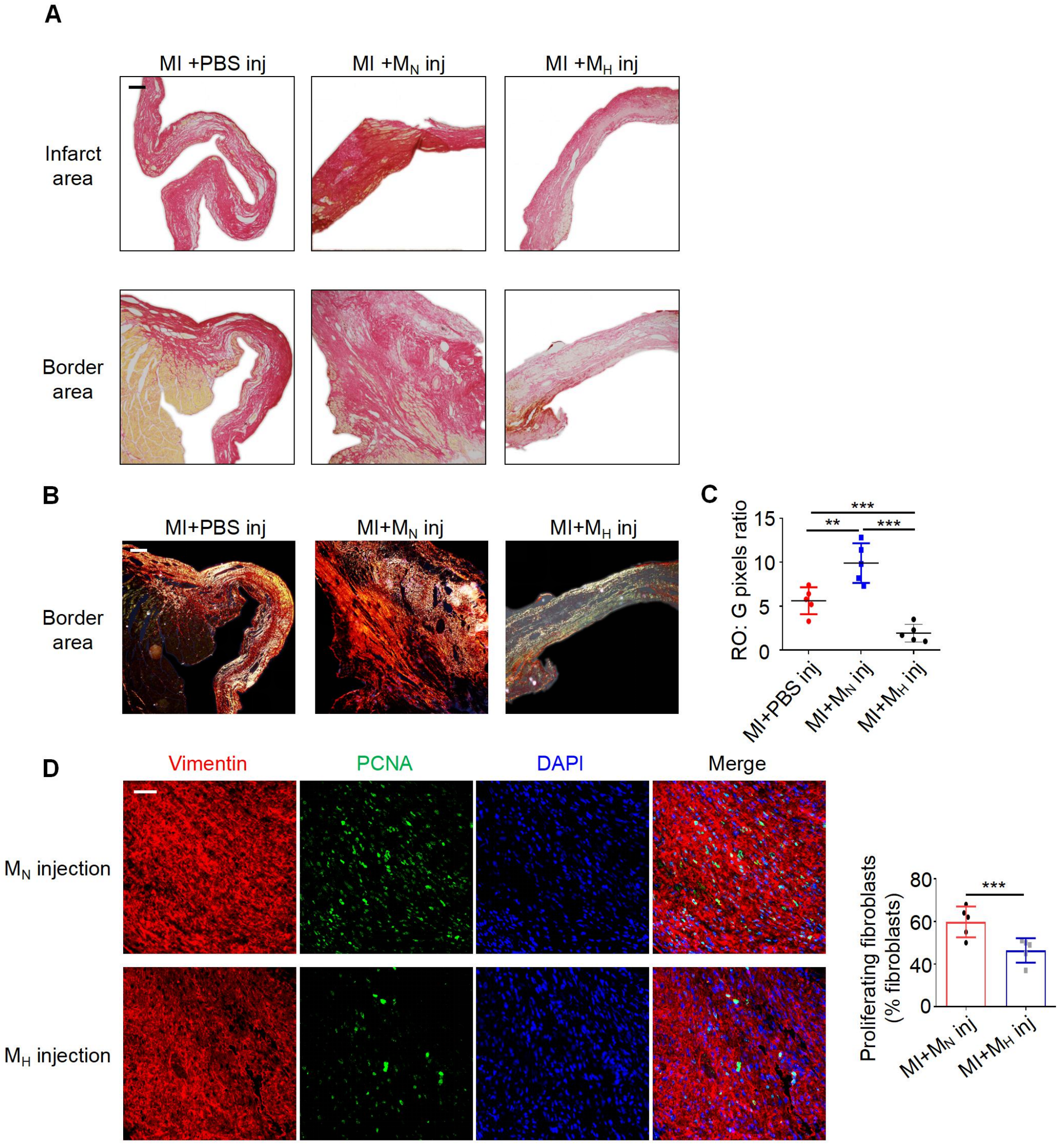

Figure S16: Hypoxia-primed monocytes/macrophages alleviated collagen deposition and LV fibrosis post-MI. (A) Representative images of infarct areas stained by picrosirius red and visualized under bright-field light. Scale bars: 100 µm. **(B)** Representative images of infarct areas stained by picrosirius red and visualized under polarised light. Scale bars: 100 µm. **(C)** Quantification of picrosirius red polarized light analysis in the infarct areas of MI mice. **(D)** Representative images of co-immunofuorescent staining and quantification of proliferating cardiac fibroblasts in the infarct area of mice under normoxia, hypoxia and hyperoxia at 7 days post-myocardial infarction (MI). Scale bars: 50 µm. Dot-plots represent data from individual mice. Statistical significance was determined by ANOVA followed by Sidak's multiple comparisons test **(C)** or unpaired *t* test **(D)**. $^{**}P < 0.$

01, ***P < 0.001 compared to control.

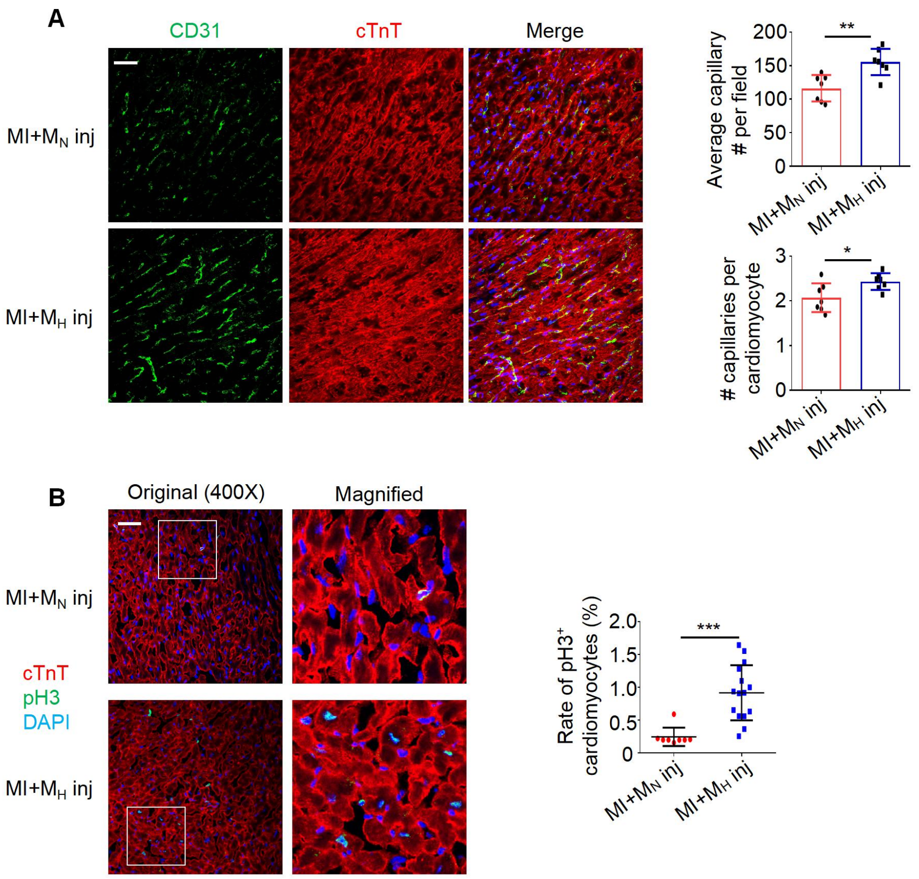

Figure S17. Hypoxia-primed monocytes/macrophages induced angiogenesis and promoted cardiomyocytes proliferation after MI. (**A**) Representative immunofluorescence images of CD31 and cTnT stained heart tissue and the quantifications after MI with macrophages injection around border zone. Scale bar: 50 μm. (**B**) Representative immunofluorescence images and its magnified pictures of pH3 and cTnT stained heart tissue after MI with macrophages injection around border zone. Scale bar: 50 μm. Dot-plots represent data from individual mice. Statistical significance was determined by unpaired *t* test (**A**, **B**). **P* < 0.05, ****P* < 0.001 compared to MI+M_N injection.

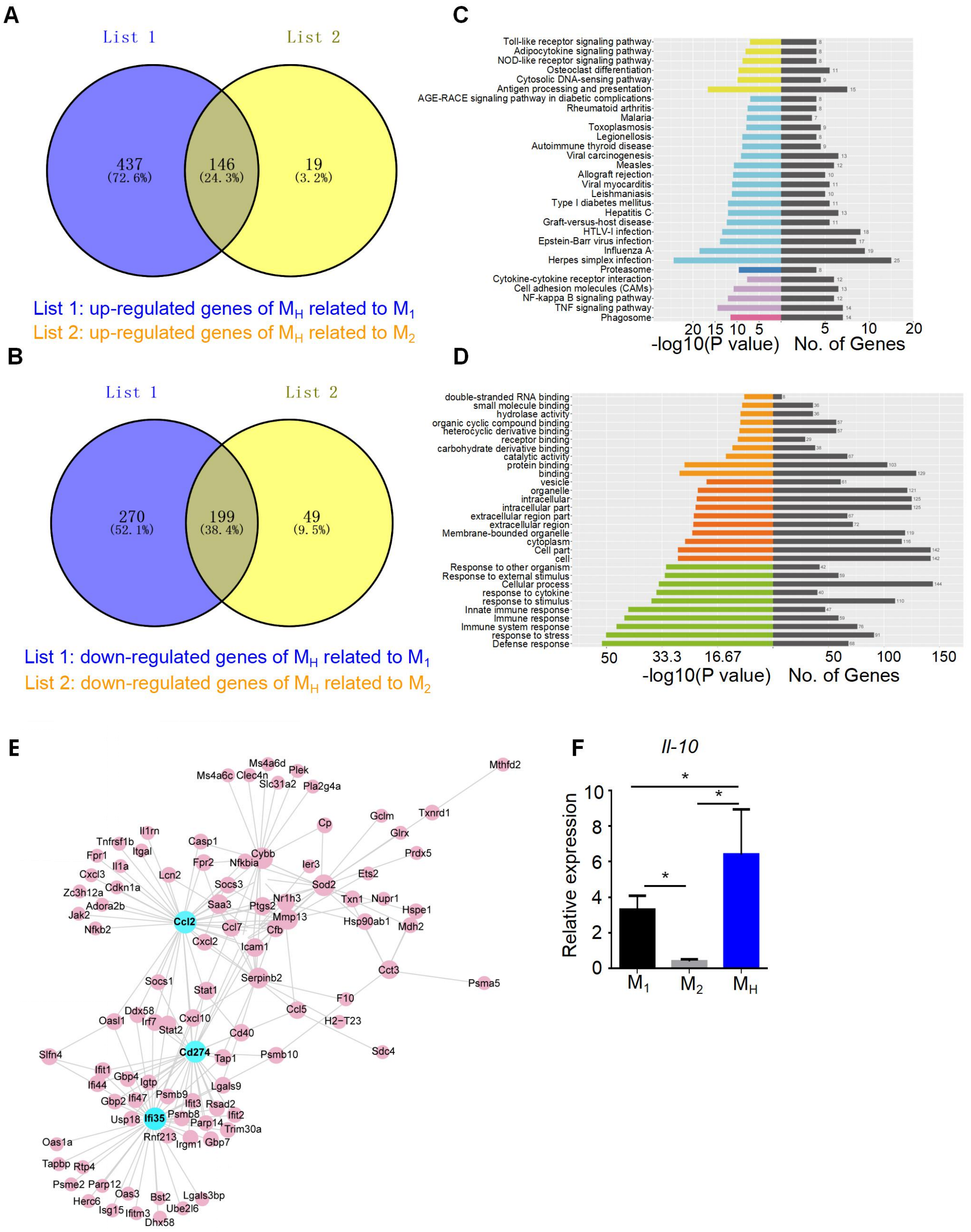

Figure S18. Genes array defining the differences between M_H macrophages relative to M_1 and M_2 macrophages. (A) Venn diagram showed 146 genes were upregulation in M_H macrophages compared to M_1 and M_2 macrophages. **(B)** 199 genes were down-regulation in M_H macrophages compared to M_1 and M_2 macrophages. **(C)** KEGG analysis of biological pathways for M_H macrophages down-regulated genes relative to M_1 and M_2 macrophages. **(D)** Functional enrichment analysis of M_H macrophages down-regulated genes relative to M_1 and M_2 macrophages. **(E)** Protein interaction network of M_H macrophages down-regulated genes relative to M_1 and M_2 macrophages. **(F)** Gene expression of IL-10 in M_1 and M_2 and M_H

macrophages relative to M_N macrophages. Statistical significance was determined by Kruskal-Wallis test, followed by a Dunn's post-test to correct for multiple comparisons. *$P < 0.05$ compared to M_N macrophages.

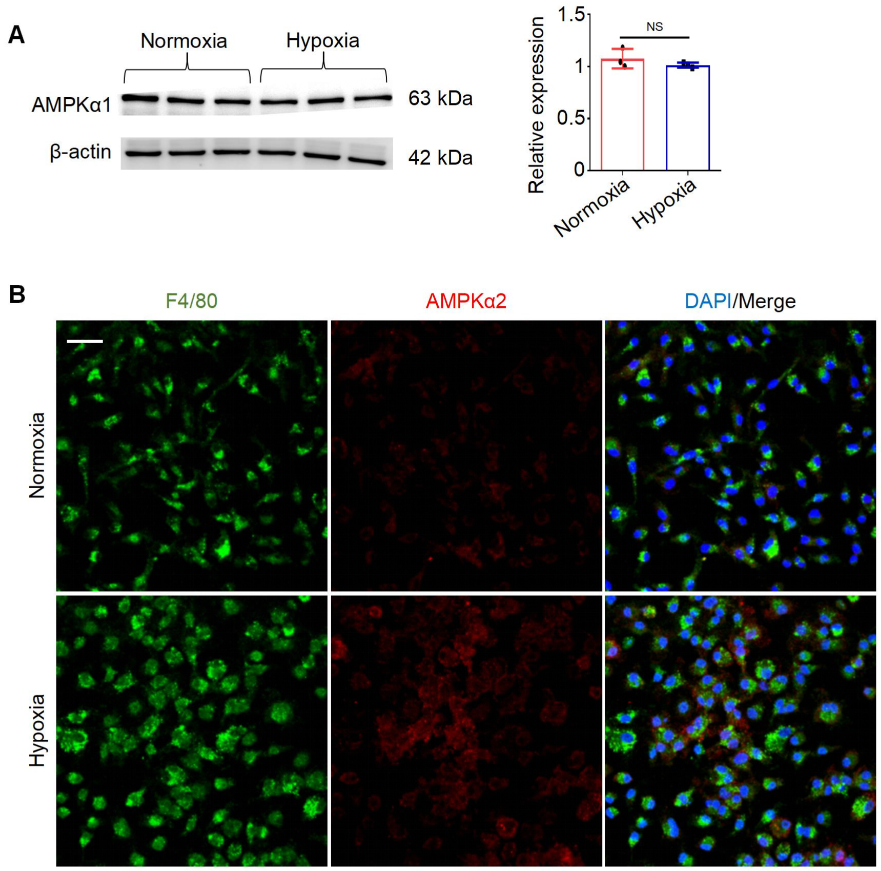

Figure S19. Expression of AMPK protein in M_H macrophages. (A) Representative protein immunoblots of AMPK α1 and pooled data from immunoblots. **(B)** Representative immunofluorescence images of AMPK α2 stained macrophages. Scale bar: 50 μm. Dot-plots represent data from individual mice. Statistical significance was determined by Mann–Whitney test. $^{NS}P > 0.05$ compared to normoxia control.

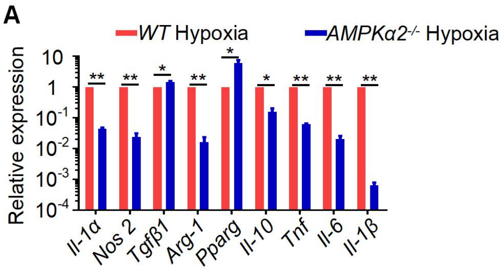

Figure S20. Macrophages were polarized by hypoxia through the AMPKα2 signaling pathway. **(A)** Gene expression profiles of hypoxic macrophages from WT and AMPKα2$^{-/-}$ mice (n = 3). Statistical significance was determined by Mann-Whitney test. **P* < 0.05, ***P* < 0.01 compared to WT hypoxia mice.

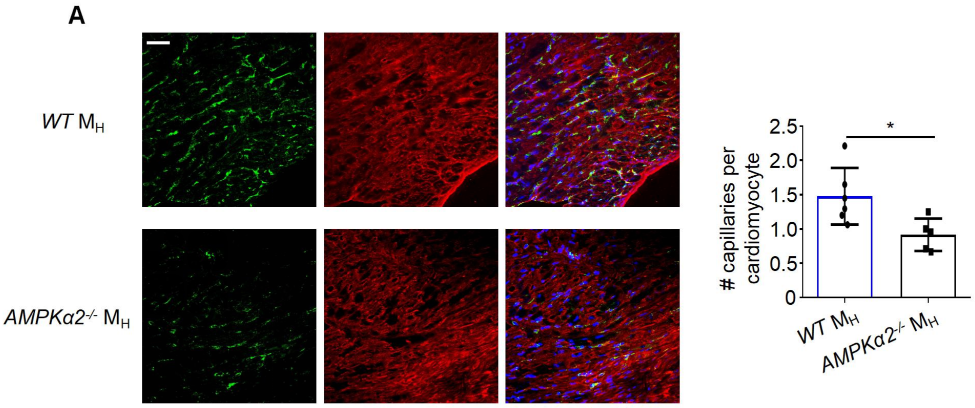

Figure S21. AMPKα2$^{-/-}$ M$_H$ macrophages transfer had negligible effect on cardiac repair after MI. (A) Representative immunofluorescence images of CD31 and cTnT stained heart tissue and the quantification after MI with macrophages injection around border zone. Scale bar: 50 μm. Dot-plots represent data from individual mice. Statistical significance was determined by unpaired *t* test. *$P < 0.05$ compared to control group.

Supplementary Tables

Table S1: Echocardiographic parameters in MI mice treat with normoxia, hypoxia, hyperoxia and sham group.

	**Post-MI**			
	**Sham**	**MI+Normoxia**	**MI+Hypoxia**	**MI+Hyperoxia**
**IVS;d;mm**	1.05±0.07	0.89±0.18	0.94±0.16	0.59±0.18
**LVID;d;mm**	3.43±0.37	5.75±0.64	4.39±0.17	5.64±0.34
**LVPW;d;mm**	0.97±0.11	0.81±0.10	0.80±0.15	0.61±0.16
**IVS;s;mm**	1.41±0.12	1.09±0.21	1.33±0.23	0.66±0.22
**LVID;s;mm**	2.48±0.30	4.78±0.38	3.33±0.18	4.96±0.54
**LVPW;s;mm**	1.28±0.11	1.16±0.42	1.03±0.09	0.82±0.29
**LV Vol;d; μl**	49.46±12.61	165.65±42.38	87.33±8.01	157.29±22.58
**LV Vol;s; μl**	22.36±6.74	107.34±19.96	45.42±5.99	117.44±25.97
**EF;%**	54.83±6.66	34.14±5.85	47.86±6.55	27.46±7.91
**FS;%**	27.83±4.21	16.59±3.31	24.01±3.88	13.09±5.17
**LV Mass;mg**	128.33±12.35	243.42±91.49	154.97±36.07	153.92±38.36
**LV Mass Corrected;mg**	102.67±9.88	194.73±73.19	123.97±28.86	123.14±30.69
**HR; n**	483.10±44.50	477.77±44.82	482.13±43.77	460.27±47.53

d, End-diastole; s, End-systole; IVS, interventricular septum; LVID, left ventricular internal dimension; LVPW, left ventricular posterior wall; LV Vol, left ventricular volume; EF, ejection fraction; FS, fractional shortening; HR, heart rate. Data are presented as means ± SD.

Table S2: Echocardiographic parameters in MI mice treat with M_H macrophages transfer.

Post-MI				
	**Sham**	**MI+PBS**	**MI+M_N**	**MI+M_H**
**IVS;d;mm**	1.12±0.28	0.91±0.16	1.10±0.11	1.04±0.08
**LVID;d;mm**	3.75±0.13	5.40±0.93	4.91±0.32	4.69±0.39
**LVPW;d;mm**	1.06±0.16	1.00±0.23	1.12±0.15	1.16±0.22
**IVS;s;mm**	1.62±0.33	1.32±0.49	1.48±0.09	1.69±0.56
**LVID;s;mm**	2.48±0.22	4.42±0.77	3.90±0.28	3.21±0.73
**LVPW;s;mm**	1.46±0.28	1.26±0.29	1.45±0.25	1.52±0.28
**LV Vol;d; µl**	60.08±5.00	146.76±57.99	113.69±17.09	102.99±20.14
**LV Vol;s; µl**	22.19±4.94	92.08±35.90	66.36±11.34	47.93±9.04
**EF;%**	62.95±7.95	37.25±7.61	41.73±2.45	52.85±7.03
**FS;%**	33.73±5.82	18.22±4.18	20.51±1.38	27.31±4.80
**LV Mass;mg**	167.98±51.70	267.05±147.68	258.24±50.14	235.98±53.50
**LV Mass Corrected;mg**	134.38±41.36	213.64±118.14	206.59±40.11	188.79±42.80
**HR, n**	462.10±46.37	465.69±52.65	476.75±45.99	448.40±63.88

d, End-diastole; s, End-systole; IVS, interventricular septum; LVID, left ventricular internal dimension; LVPW, left ventricular posterior wall; LV Vol, left ventricular volume; EF, ejection fraction; FS, fractional shortening; HR, heart rate. Data are presented as means ± SD.

Table S3: Primary antibody lists

**Primary antibody**	**Host species**	**Supplier**
**Anti-pH3**	Rabbit	Millipore
**Anti-Aurora B**	Rabbit	Abcam
**cTnT**	Mouse	Abcam
**CD31**	Rabbit	Abcam
**F4/80**	Rat	Abcam
**AMPKα2**	Rabbit	Abcam
**AMPKα1**	Rabbit	Abcam
**CCR2**	Mouse	Abcam
**HiF-1α**	Rabbit	Abcam
**CD68**	Rabbit	Proteintech
**CD206**	Rabbit	Proteintech

Table S4: Flow cytometry primary antibody.

Flow cytometry primary antibody	Format	Clone
Anti-mouse **CD45** antibody	APC	30-F11
Anti-mouse **F4/80** antibody	PE/Cy7	BM8
Anti-mouse **I-Ab** antibody	FITC	AF6-120.1
Anti-mouse **Ly-6G** antibody	PerCP/Cy5.5	1A8
Anti-mouse **CCR2** antibody	PE	475301
Anti-mouse/human **CD11b** antibody	APC	M1/70
Anti-mouse **CD19** antibody	PE	AFS98
Anti-mouse **CD115** antibody	PE	HI98
Anti-mouse **Ly-6G** antibody	APC/Cy7	1A8
Anti-mouse **F4/80** antibody	FITC	BM8
Anti-mouse **I-Ab** antibody	PerCP/Cy5.5	AF6-120.1
Anti-mouse **Ly-6C** antibody	PE/Cy7	HK1.4
Anti-mouse **Lineage Cocktail** with Isotype Ctrl	FITC	145-2C11;RB6-8C5; RA3-6B2; Ter-119; M1/70;
Anti-mouse **Sca-1** antibody	PerCP/Cy5.5	D7
Anti-mouse **c-kit** antibody	PE/Cy7	104D2
Anti-mouse **CD68** antibody	PerCP Cy5.5	FA-11
Anti-mouse **CD206** antibody	BV421	C068C2
Anti-mouse **CD16/32** antibody	APC	93

Table S5: Primer names and sequences of the inflammatory factors.

Primer Name	Froward sequence 5' to 3'	Reverse sequence 5' to 3'
CCL2	TTTTTGTCACCAAGCTCAAGAG	TTCTGATCTCATTTGGTTCCGA
CCL5	GTATTTCTACACCAGCAGCAAG	TCTTGAACCCACTTCTTCTCTG
CXCL1	CCGCTCGCTTCTCTGTGCAG	ACTGACAGCGCAGCTCATTGG
FASLG	CAGTTTTTCCCTGTCCATCTTG	CCTTCTTCTTTAGAGGGGTCAG
IFN-γ	CTTGAAAGACAATCAGGCCATC	CTTGGCAATACTCATGAATGCA
IL-13	ACCCTTAAGGAGCTTATTGAGG	ATTGCAATTGGAGATGTTGGTC
L-selectin	TGCCCAAAAGCCCTTATTACTA	CTCCTTGGACTTCTTGTTGTTG
MIP-3α	TCTTCCTTCCAGAGCTATTGTG	GACTGCTTGTCCTTCAATGATC
MMP8	TTGAGAAAGCTTTTCACGTCTG	CTTGAGACGAAAGCAATGTTGA
CXCL7	ACGAATACCATCTCTGGAATCC	TTCTTCAGTGTGGCTATCACTT

Table S6: Primer names and sequences of the macrophage canonical markers.

Primer Name	Froward sequence 5' to 3'	Reverse sequence 5' to 3'
IL-1a	ATCAGCAACGTCAAGCAACG	GGTTGGATGGTCTCTTCCAGA
NOS2	TGATGTGCTGCCTCTGGTCT	GAGCTCCTGGAACCACTCGT
TNF	ACTGGCAGAAGAGGCACTCC	CTGCCACAAGCAGGAATGAG
Arg-1	CAATGAAGAGCTGGCTGGTG	GGCCAGAGATGCTTCCAACT
Pparg	GGACATCCAAGACAACCTGCT	TGTGACGATCTGCCTGAGGT
TGFβ1	GCGGCAGCTGTACATTGACT	ACTGTGTGTCCAGGCTCCAA
IL-6	TCCATCCAGTTGCCTTCTTG	AAGCCTCCGACTTGTGAAGTG
IL-10	CACTGCTATGCTGCCTGCTC	GAAGGCAGTCCGCAGCTCTA
Il-1β	CCAGGATGAGGACATGAGCA	CGGAGCCTGTAGTGCAGTTG
